# Microenvironmental signals combine to induce non-additive molecular and phenotypic responses in mammary epithelial cells

**DOI:** 10.1016/j.isci.2025.113407

**Published:** 2025-08-20

**Authors:** Ian C. McLean, Sean M. Gross, Jeremy Copperman, Daniel S. Derrick, Indranil Paul, Andrew Emili, Laura M. Heiser

**Affiliations:** 1 Department of Biomedical Engineering, OHSU, Portland, OR, USA; 2 Capsida Biotherapeutics Inc., Thousand Oaks, CA, USA; 3 Cancer Early Detection Advanced Research Center, OHSU, Portland, OR, USA; 4 Knight Cancer Institute, OHSU, Portland, OR, USA; 5 Division of Oncological Sciences, OHSU, Portland, OR, USA

**Keywords:** Microenvironment, Systems biology, Cell biology

## Abstract

Cellular phenotypes are dictated not by single signals but by the integration of multiple extracellular cues, a process that remains poorly understood. Here, we systematically dissect how combinations of Oncostatin M, Transforming Growth Factor β1, and Epidermal Growth Factor shape the behavior of MCF10A mammary epithelial cells. Live-cell imaging revealed that ligand combinations drive emergent phenotypes absent in single-ligand contexts, pointing to the induction of novel molecular programs. Transcriptomic profiling uncovered synergistically regulated genes, while partial least-squares regression linked these transcriptional signatures to quantitative imaging-derived phenotypes and validated them across independent datasets. Functional analysis revealed CXCR2 signaling, upregulated through CREB activation, as a key driver of enhanced cell motility under combinatorial ligand treatment. Together, these findings establish a framework for uncovering how extracellular signals converge to modulate gene expression and phenotype, providing new insight into the principles governing complex epithelial cell behaviors.

## Highlights

• Combination ligand treatments produce emergent phenotypes
• Ligand combinations drive both synergistic and repressive transcriptional changes
• Partial least-squares regression predicts phenotypic responses from transcriptomic data
• CXCR2 activation promotes synergistic motility in EGF+OSM-treated epithelial cells

## Graphical abstract

**Figure:**
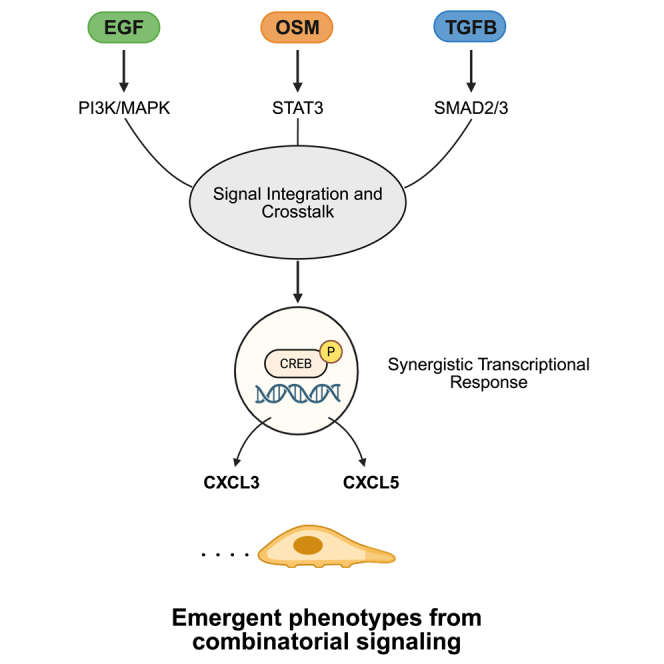

## Introduction

The intricate interplay between cells and their microenvironment is a fundamental determinant of cellular behavior and function. Central to this dynamic relationship is the diverse array of ligands, cytokines, and extracellular matrix proteins that can initiate myriad intracellular responses that ultimately shape cellular phenotype. Extracellular signals play essential roles in guiding development and maintaining normal tissue function, orchestrating processes such as differentiation, proliferation, and migration.^1^ Conversely, the deregulation of extracellular signals leads to aberrant cell-cell interactions, which can drive disease.^2^ While the impacts of individual extracellular signals have been extensively studied, the understanding of how cells integrate multiple extracellular signals into a cohesive response is limited.

In this study, we investigate the combinatorial effects of three ligands—Oncostatin M (OSM), Transforming Growth Factor β 1 (TGFB), and Epidermal Growth Factor (EGF)—on the phenotypic and molecular responses of MCF10A mammary epithelial cells. These ligands hold pivotal roles in the development and function of mammary tissue, and their dysregulation is associated with disease.^3^^,^^4^^,^^5^ EGF canonically activates the MEK/ERK and PI3K signaling pathways^6^; OSM signals through JAK/STAT^7^; and TGFB orchestrates SMAD-mediated processes.^8^ The signaling pathways activated by EGF regulate the motility, proliferation, and invasion of normal and malignant breast epithelial cells.^6^ TGFB induces epithelial-to-mesenchymal transition (EMT) in breast epithelial cells, achieved through the induction of EMT-associated transcription factors including SNAI1 and SNAI2, resulting in stereotyped changes in cell morphology and motility.^9^ TGFB also influences breast epithelial cell proliferation by activating p21 and suppressing key cell cycle transcription factors such as MYC, leading to cell-cycle arrest.^10^ The activation of JAK/STAT signaling downstream of OSM and other IL6 family cytokines modulates invasive properties and alters motility.^11^^,^^12^ Despite their well-defined molecular consequences, the interplay between these signaling pathways and their combined impact on cellular behavior remains poorly understood.

Although the effects of these individual ligands have been examined in various cellular contexts,^11^^,^^13^^,^^14^^,^^15^^,^^16^ predicting how cells will respond to dual treatment at the molecular or phenotypic level is challenging. This is due in part to the poorly defined molecular networks that integrate extracellular cues to drive phenotypic changes as well as the inherent complexity of intracellular signaling, which involves non-linearity,^17^ feedback regulation,^18^ and extensive pathway crosstalk.^19^ These factors can produce emergent behaviors that cannot simply be inferred from single-ligand responses. Previous studies on combinatorial perturbations have mainly focused on exploring and predicting interactions between therapeutic inhibitors with a focus on synergy—the concept that the interaction between two inhibitors induces an effect that is greater than the sum of the individual effects of each drug. Computational modeling approaches have demonstrated that synergistic drug interactions can be partially predicted from the transcriptional profiles of cells treated with single drugs.^20^^,^^21^^,^^22^^,^^23^ In addition, there is a strong correlation between synergistic gene expression patterns and the degree of drug synergy, demonstrating a robust association between combinatorial transcriptional dynamics and resultant phenotypic responses.^24^ To date, the exploration of combinatorial effects and relationships between transcription and phenotype has typically been confined to a single phenotypic response, and primarily focused on viability. However, there remains a critical unmet need to systematically investigate how combinatorial perturbations are encoded to mediate a broader range of phenotypic outcomes.

Employing live-cell imaging and RNA sequencing, we systematically investigated the phenotypic and transcriptional responses of MCF10A mammary epithelial cells after treatment with EGF, TGFB, OSM and combinations thereof. MCF10A is a well-characterized model system that has been utilized extensively to investigate tissue development, migration, and proliferation.^25^^,^^26^^,^^27^^,^^28^^,^^29^ We found that all combinations of ligands induce phenotypic responses that differ significantly from their respective single-ligand phenotypes, suggesting the differential activation of molecular programs. Motivated by this finding, we performed a comprehensive transcriptomic analysis to identify synergistic transcriptional programs in each combination condition, which revealed specific programs modulated in response to combination treatments. We used partial least-squares regression (PLSR)^30^ to decipher the complex relationship between transcriptional programs and cellular phenotype. Our comprehensive analysis revealed that when combined, EGF and OSM synergistically amplify molecular programs associated with leukocyte chemotaxis and CXCR2 activation, resulting in increased cell motility. Functional validation demonstrated that the synergistic upregulation of CXCR2-associated chemotactic factors is mediated by CREB transcription factor activation.

## Results

### Epidermal growth factor, oncostatin M, and transforming growth factor β 1 treatment combinations induce emergent phenotypic responses

**Figure 1:**
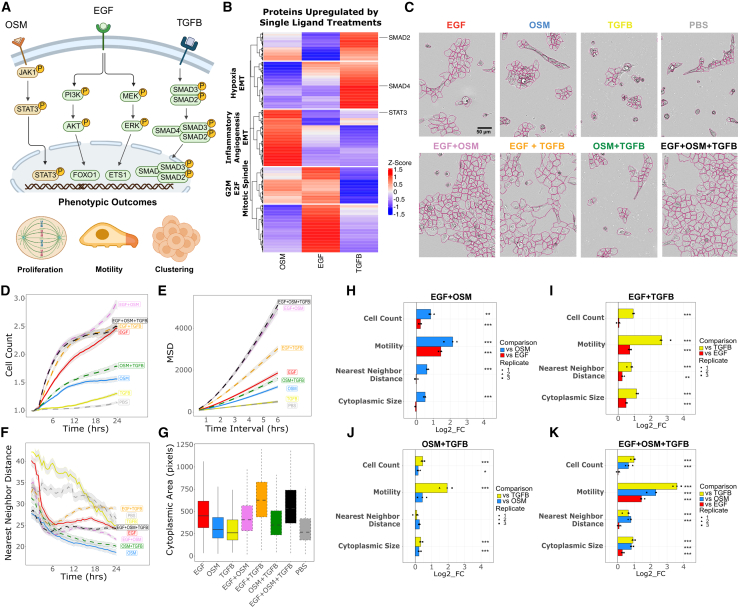
Combination treatments induce diverse and emergent phenotypic behavior MCF10A cells were treated with PBS (control), EGF, OSM, or TGFB alone, or their pairwise/triple combinations, and subjected to live-cell imaging and quantitative analysis over 24 h. (A) Overview of the signaling pathways activated by each treatment, highlighting the canonical pathways associated with each ligand. Schematic representations depict the potential phenotypic outcomes (e.g., proliferation, motility, and clustering/spreading) influenced by these pathways. (B) Heatmap showing the top 100 most upregulated proteins induced by the indicated ligand, as identified by mass spectrometry. EGF, OSM, and TGFB each induce distinct modules enriched for proliferation, inflammation, EMT, and hypoxia-related pathways (q-value <0.05). Selected proteins include SMAD2/4 (TGFB) and STAT3 (OSM). Protein intensities are *Z* score normalized across samples. (C) Representative images of MCF10A cells under different ligand treatments at 24 h, demonstrating changes in cell phenotype. Segmented cell outlines are indicated in pink (scale bar = 50 μm). (D–F) Quantification of cell phenotype from 0 to 24 h (cell count normalized to T0, MSD, nearest neighbor distance). MSD was calculated as the mean squared displacement of cell trajectories over time. Shaded region represents the 95% confidence interval of the mean. (G) Boxplots depict cytoplasmic area of segmented cells. Boxes represent the interquartile range with the median shown as a line; whiskers indicate 1.5× the IQR. (H–K) Quantified phenotypic responses for each combination condition were compared to each single-ligand condition comprising that combination. ANOVA followed by post-hoc Tukey’s honest significant difference test was used to assess significance, with *p*-value <0.05 considered significant (*n* = 3). ∗*p* < 0.05, ∗∗*p* < 0.01, ∗∗∗*p* < 0.001.

We studied responses to three ligands (EGF, OSM, and TGFB) that canonically activate distinct signaling pathways and that have been shown to induce strong phenotypic responses in MCF10A mammary epithelial cells^11^ (Figure 1A). Mass spectrometry-based proteomic analysis revealed largely orthogonal protein expression profiles 24 h following EGF, OSM, or TGFB treatment (Figure 1B; Table S1). TGFB induced a unique module enriched for EMT and hypoxia-associated proteins, including SMAD2 and SMAD4; OSM upregulated a distinct inflammatory and angiogenic module marked by STAT3; and EGF robustly activated a mitotic signaling module enriched for G2M checkpoint, E2F targets, and mitotic spindle proteins, with partial overlap with OSM. These findings are consistent with canonical signaling paradigms^7^^,^^31^^,^^32^ and provide additional evidence that each ligand activates a distinct regulatory network.

To investigate how cells integrate extracellular signals into coordinated molecular and phenotypic responses, MCF10A cells were cultured in assay medium lacking EGF and insulin, then treated with EGF, OSM, and TGFB—either individually, in pairs, or all three combined. Because EGF is normally included in MCF10A culture media,^33^ EGF-treated cells served as a positive control. Cells treated with PBS (vehicle) in assay media served as a negative control. After treatment, cells were subjected to live-cell imaging every 30 min for 24 h.^29^ We employed quantitative image analysis to assess changes in cell count, motility, nearest neighbor distance, and cytoplasmic size (Figure 1C). In our initial analyses, we describe the quantitative responses to each single ligand as compared to the PBS control. Consistent with previous studies,^11^ we found that independent treatment with EGF strongly induced proliferation, OSM showed more modest proliferative effects, while TGFB minimally influenced proliferation as compared to PBS (Figures 1D and S1A). Cell motility was evaluated by calculating the mean squared displacement (MSD) over all cells for each treatment and deriving the diffusion coefficient from the MSD curves (Figures 1E and S1B). Like cell proliferation, EGF treatment significantly increased cell motility compared to PBS control, OSM induced a modest increase in motility, and TGFB showed no change in motility compared to PBS (Figures 1E and S1A). The calculation of the distance to the nearest neighboring cell revealed changes in cell clustering (decreased neighbor distance) and cell spreading (increased neighbor distance). EGF and TGFB treatment did not significantly affect the nearest neighbor distance compared to PBS control; however, OSM led to a decrease in this metric, indicating tight cell clustering (Figures 1F and S1A). Lastly, we observed that EGF treatment increased cytoplasmic size compared to the PBS control, whereas TGFB or OSM alone had no effect (Figures 1G and S1A). In total, these analyses demonstrate that each treatment regimen induced a unique constellation of phenotypic changes.

Given the distinct phenotypic responses associated with each ligand, we hypothesized that combination treatment responses would adhere to the Highest Single Agent (HSA) model.^34^ The HSA model, used to study drug combination synergy, posits that the expected phenotypic response for a combination treatment is equal to the maximal effect of any single agent. Using this framework, we predicted that the phenotypic responses to ligand combinations would primarily reflect the influence of the single ligand with the most significant impact on each aspect of cell phenotype. To test this hypothesis, we compared phenotypic metrics measured in each combination condition to those of each respective single-ligand condition. A phenotype was considered emergent (i.e., it did not mirror either single-ligand response) if we detected a significant change that deviated from each single-ligand condition, assessed using a one-way analysis of variance (ANOVA) followed by post-hoc Tukey’s honest significant difference test (*p*-value <0.05).

Consistent with the HSA model, cell count observed under EGF+TGFB and EGF+OSM+TGFB treatments remained unchanged compared to EGF alone. However, EGF+OSM and OSM+TGFB treatments resulted in a cell count greater than either of the individual ligand treatments (Figures 1H–1K). This finding suggests an interaction between OSM and the other ligands that enhances cell proliferation. We also observed emergent phenotypic changes in cell motility, distance to nearest neighboring cells, and cytoplasmic size in combination treatments. Treatment with OSM and TGFB individually resulted in decreased cell motility as compared to EGF treated cells (Figure S1C). However, combination treatment with EGF+OSM, EGF+TGFB, and EGF+OSM+TGFB resulted in an emergent increase in cell motility compared to respective single-ligand conditions (Figures 1H, 1I, and 1K).Finally, combination treatment with EGF+TGFB resulted in an emergent phenotypic increase in both nearest neighbor distance and cytoplasmic size, indicating cell spreading (Figure 1I). The addition of TGFB to EGF increased cytoplasmic size beyond that induced by EGF alone, mimicking morphological and cytoskeletal changes characteristic of mesenchymal-like transition^35^ (Figure S1C). Our findings indicate that across various ligand combination treatments and multiple phenotypic responses, combination treatments induce emergent cellular phenotypes that diverge from individual ligand treatments, suggesting that combination treatments induce molecular programs not observed after single-ligand treatments.

To dissect the role of canonical downstream signaling pathways MEK and PI3K in driving motility and proliferation responses,^36^^,^^37^ we applied selective inhibitors to assess pathway activity across single- and combination-ligand treatments. We observed distinct pathway dependencies across ligand conditions, particularly in cell proliferation. All OSM-containing conditions showed reduced reliance on MEK signaling for maintaining cell count, whereas EGF-containing conditions in the absence of OSM (EGF, EGF+TGFB), exhibited greater reliance on MEK signaling (Figure S1D). Conversely, certain OSM-induced responses (OSM, EGF+OSM) were more strongly dependent on PI3K signaling, while conditions containing EGF and TGFB (EGF+TGFB, EGF, TGFB) showed less sensitivity to PI3K inhibition. Notably, in the triple combination treatment (EGF+OSM+TGFB), the activation of multiple pathways appeared to reduce proliferative reliance on MEK signaling, suggesting that combination treatments engage additional signaling networks to drive proliferation. In contrast, motility was more uniformly reliant on MEK signaling across all conditions (Figure S1E). PI3K inhibition led to a stronger reduction in motility than MEK inhibition across most conditions, consistent with the role of PI3K signaling in cytoskeletal dynamics.^38^ However, we also observed ligand-dependent effects, as TGFB and OSM+TGFB exhibited only a modest decrease in motility following the inhibition of either pathway. These findings confirm that ligand treatments differentially utilize MEK and PI3K signaling pathways, leading to distinct effects on cell proliferation and motility depending on the specific ligand context.

### Ligand combinations modulate and reprogram transcription factor networks

**Figure 2:**
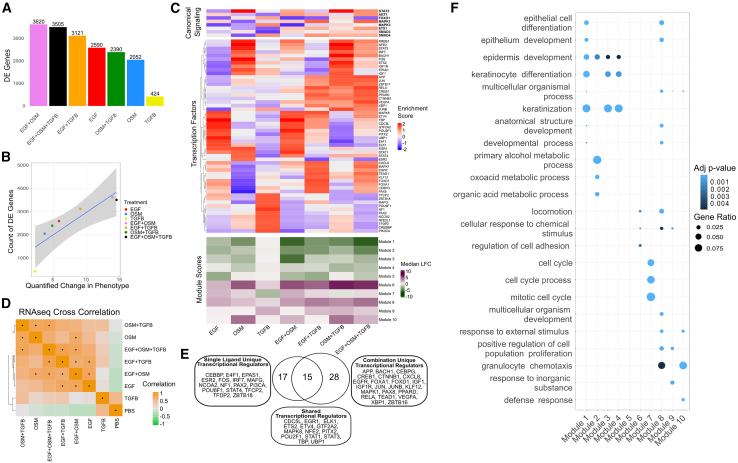
Transcriptional programs induced by single and combination ligand treatments (A) Number of differentially expressed genes (LFC >1.5 or < −1.5, q-value <0.05) across treatments relative to PBS control, showing the greatest response in EGF+OSM treatment. (B) Correlation between the number of differentially expressed genes and overall changes in cell phenotype (R^2^ = 0.75, *p* = 0.012). Shaded region represents the 95% confidence interval. (C) Enrichment analysis of canonical transcriptional regulators (Canonical Signaling, top heatmap), the top decile of transcription factors (Transcription Factors, middle heatmap), and median log fold change (LFC) for gene modules (Module Score, bottom heatmap) activated in response to each treatment condition. (D) Pairwise Pearson correlations of gene expression log fold changes compared to T0 between all treatments. Correlations >0.7 are indicated with a dot. (E) Venn diagram comparing transcriptional regulators enriched in single versus combination treatments. (F) Enrichment of Gene Ontology terms for each gene module identified. Dot size represents the gene ratio calculated as the proportion of genes in a given module associated with a specific GO term. Dot color indicates the adjusted p-value, corrected using the g:SCS method.

We next used RNA sequencing to examine the molecular mechanisms driving phenotypic responses to ligand combinations. Cells were harvested after 24 h of ligand treatment and subjected to bulk RNA sequencing. We posited that treatments leading to the strongest changes in cell phenotype compared to the PBS control would likewise display the greatest change in transcriptional responses. To test this hypothesis, we first assessed the overall transcriptional perturbation for each ligand condition by quantifying the number of differentially expressed genes as compared to PBS control (LFC >1.5 or LFC < −1.5 and q-value <0.05). Treatments modulated gene expression programs to different extents; the EGF+OSM treatment induced the greatest number of differentially expressed genes, while independent treatment with TGFB had only modest impact on transcription (Figures 2A, S2A, and S2B). To assess the overall change in cell phenotype for each condition, we calculated the phenotypic response magnitude: the total magnitude of the four phenotypic metrics relative to PBS control. This was done by representing the phenotypic metrics as a four-dimensional vector, where the origin indicates no change from PBS control, and computing the phenotypic response magnitude as the vector magnitude across these four dimensions. Then, for each treatment, we directly compared the phenotypic response magnitude to the number of differentially expressed genes (Figure 2B). We found a strong correlation between the number of differentially expressed genes and the phenotypic response magnitude (R^2^ = 0.75, *p*-value = 0.012). The treatments that provoked the most significant changes in gene expression also corresponded to the most substantial overall changes in cell phenotype (EGF+OSM+TGFB, EGF+OSM, and EGF+TGFB). Conversely, treatments that exerted only modest changes in cell phenotype were associated with the fewest changes in gene expression, highlighting a clear relationship between gene expression alterations and phenotypic outcomes across different treatment conditions.

Given that transcription factors (TFs) are critical regulators of gene expression programs, we sought to identify the TFs most strongly modulated by single and combination treatments. We performed transcription factor enrichment on the RNA-seq dataset using Priori, an approach that leverages prior biological information to infer the activity of transcriptional regulators.^39^ Priori evaluates the activation levels of canonical transcription factors and enables the data-driven identification of key signaling proteins that may play a crucial role in mediating ligand responses. Analysis of the relative enrichment of canonical transcriptional regulators in response to single-ligand treatments recapitulates established signaling pathways associated with each ligand. Namely, EGF enriches AKT and MAPK transcriptional processes, OSM induces STAT3, while TGFB induces SMAD4 programs, respectively (Figure 2C, Canonical Signaling). Relative to EGF alone, EGF+TGFB increases MAPK enrichment, and EGF+OSM increases AKT enrichment. These findings are consistent with the inhibitor experiments described above, where the EGF+OSM treatment exhibited greater proliferative reliance on PI3K signaling, while EGF+TGFB treatment induced proliferation primarily through MEK signaling (Figure S1D). Conversely, EGF+OSM diminishes STAT3 enrichment relative to the OSM condition (Figure 2C, Canonical Signaling). We also compared the top decile of transcription factors most strongly induced by each treatment condition (Figure 2C, Transcription Factors), which revealed that approximately half of the transcriptional regulators enriched in single-ligand treatments are also active in their respective combination conditions (16/33) (Figures 2E and S2C). However, almost two-thirds (64%, 28/44) of transcriptional regulators are enriched only in combination treatment conditions, including growth factor signaling molecules (EGFR, IGF1, IGF1R, MAPK1, VEGFA) and regulators of epithelial and immune cell differentiation (APP, BACH1, CEBPG, CREB1, CTNNB1, RELA). This may indicate that a common molecular program is induced in response to treatment with multiple ligands.

### Combination treatments are encoded through the modulation of single-treatment transcriptional programs

We next sought to identify coordinated molecular programs associated with the observed phenotypic responses. Focusing on the subset of genes most strongly induced relative to the T0 control, we identified the top 200 most up-regulated and top 200 most down-regulated genes for each treatment (*p*-value <0.05, LFC >1.5). Pairwise correlation of the log fold changes revealed that combination treatments share transcriptional similarities with at least one ligand in each pair (Pearson correlation >0.7) (Figure 2D). To further explore the molecular mechanisms driving the distinct phenotypic responses in the combination conditions, we applied K-means clustering^40^ and gap analysis,^41^ which identified 10 coordinated gene modules (Figure S2D). Gene module scores, calculated as the median expression for each module, were not exclusively up- or down-regulated in any combination condition, suggesting that combination responses modulate existing transcriptional programs (Figure 2C, Module Scores).

Gene set enrichment analysis (GSEA) revealed that transcriptional modules identified in our dataset correspond to distinct molecular programs, with several modules aligning with pathways associated with epithelial differentiation, cell cycle regulation, and motility (Figure 2F).^42^^,^^43^^,^^44^ Gene modules 1, 2, 3, and 4 are enriched for terms related to epithelial cell and keratinocyte differentiation and development. These modules are consistently downregulated across all treatments, except for the TGFB condition. The downregulation of differentiation-associated modules in the EGF+OSM and EGF+OSM+TGFB conditions is notable, as these treatments also induced the most significant increase in cell motility (Figures S2E and S2H). This suggests that the loss of epithelial differentiation programs may be linked to the promotion of cell motility. On the other hand, the preservation of these modules in the TGFB condition supports its role as a regulator of differentiation, directing the balance between differentiation and motility in response to disparate extracellular signals (Figures 1C and 1D).

Further exploration of the molecular programs associated with cell cycle and motility revealed distinct expression patterns of the gene modules. Module 7, enriched for cell cycle-related terms, shows the highest expression under the EGF and EGF+OSM conditions, correlating with the increased cell count observed in these treatments. Modules 6 and 8, enriched for terms related to general cell motility (locomotion), were upregulated across various conditions, with module 6 showing the greatest upregulation in EGF+TGFB, OSM+TGFB, and EGF+OSM+TGFB combinations. This suggests that these conditions amplify general motility-related transcriptional programs, consistent with the enhanced cell motility observed (Figures S2F–S2H). Conversely, module 10, which is enriched for granulocyte chemotaxis terms, is specifically upregulated in conditions containing OSM. This indicates that OSM plays a unique role in activating specific chemotaxis pathways that may be critical for cell motility in response to specific combination treatments. Together, these findings suggest that ligand combinations modulate existing regulatory networks, altering differentiation, proliferation, and motility programs in ways that correspond to the phenotypic behaviors observed in live-cell imaging. However, the emergence of previously unengaged transcription factors in combination treatments suggests that these conditions may initiate additional transcriptional programs not fully captured by this high-level analysis.

### Combination treatments induce synergistic transcriptional programs

In previous sections, we demonstrated that combination conditions engage unique transcriptional regulators not observed in single-ligand treatments. To further investigate how these transcriptional changes emerge, we evaluated whether the global gene expression patterns in combination treatments could be explained by a simple additive model. Prior studies suggest that upregulated gene responses to ligand combinations often follow either additive or multiplicative patterns.^45^ To test whether this applies to our dataset, we modeled the expected expression in combination conditions by summing the fold changes (relative to T0) of upregulated genes (LFC >0.5) from single-ligand treatments. We then compared these predicted values to observed gene expression levels in the combination conditions. This analysis revealed a strong correlation between predicted and actual expression across all ligand combinations (EGF+OSM - R^2^ = 0.73, *p*-value <0.001; EGF+TGFB - R^2^ = 0.70, *p*-value <0.001; OSM+TGFB - R^2^ = 0.76, *p*-value <0.001; EGF+OSM+TGFB - R^2^ = 0.66, *p*-value <0.001), indicating that at the global level, ligand combination responses predominantly follow an additive transcriptional model (Figures S3A–S3D).

**Figure 3:**
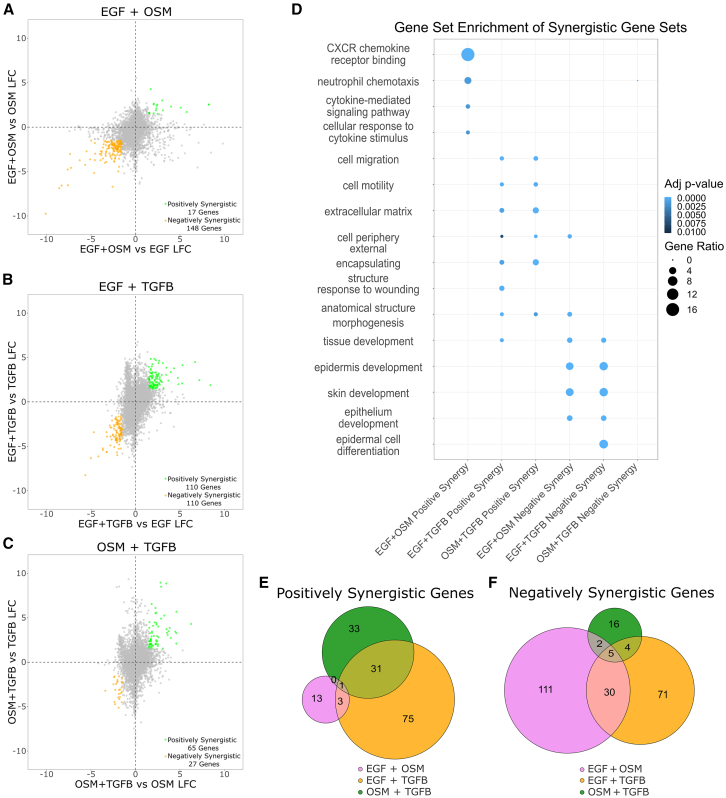
Synergistic transcriptional programs induced by ligand combination treatments (A–C) Scatterplots showing log fold change in gene expression for combination treatments versus each respective single-ligand condition (EGF+OSM, EGF+TGFB, OSM+TGFB). Genes exhibiting positive synergy (LFC >1.5, adjusted *p*-value <0.05 compared to both treatments) are shown in green, and genes with negative synergy (LFC < −1.5, adjusted *p*-value <0.05 compared to both treatments) are shown in orange. (D) Gene Ontology enrichment analysis of positively and negatively synergistic gene sets. (E) Venn diagram showing overlap of positively synergistic genes between combination conditions. (F) Venn diagram depicting negatively synergistic genes between combination conditions.

We identified 28 transcription factors uniquely enriched in combination conditions, indicating regulatory elements that become active only when multiple signaling pathways are engaged simultaneously. These transcription factors may mediate key aspects of the emergent phenotypic effects observed in combination conditions, aligning with prior studies showing that modest transcriptional synergy can have substantial functional consequences,^24^ and leading to the hypothesis that emergent phenotypic effects arise from specific synergistic transcriptional patterns not captured in our previous analysis. Borrowing ideas from drug combination studies, we applied HSA modeling to identify synergistic gene expression programs by comparing the observed response of a combination treatment to the maximum response of the individual treatments.^34^ For each combination treatment, we calculated the log fold change in expression for all genes compared to each respective single-ligand condition, then visualized the expression changes in x-y scatterplots (Figures 3A–3C). We designated a gene as positively synergistic if the log fold change in expression after combination treatment exceeded 1.5 compared to each single-ligand condition (adjusted *p*-value <0.05) (Figures 3A–3C). Similarly, we defined negatively synergistic genes as those with a log fold change of at least −1.5 compared to both respective single-ligand conditions. This analysis revealed that for each pairwise combination, subtle, yet significant, synergistic transcriptional programs are induced. The number of positively synergistic genes per combination condition ranged from 17 to 110, while negatively synergistic genes ranged from 27 to 148.

To contextualize these findings, we applied the same synergy analysis to an independent dataset of MCF7 breast epithelial cells treated with Tamoxifen, Mefloquine, and Withaferin individually and in combination (Figures S3E–S3G).^24^ These drug combinations were previously shown to exhibit phenotypic synergy by significantly reducing cell viability. We found that the degree of transcriptional synergy in our ligand combinations (17–110 genes) was comparable to two of the drug combinations (50 genes, 137 genes) but substantially lower than the highly synergistic Mefloquine-Tamoxifen treatment (2235 genes). Interestingly, even in the Mefloquine-Withaferin condition, which exhibited relatively weak transcriptional synergy, strong phenotypic synergy was observed. This suggests that while the number of synergistically regulated genes in our ligand combinations is limited, their functional impact may be significant.

We performed gene set enrichment analysis of the positive and negative synergistic gene sets identified for each combination treatment using Gene Ontology Biological Process, Molecular Function, and Cellular Component gene sets.^42^^,^^43^^,^^44^ Positively synergistic genes induced by the combination of EGF and OSM upregulated chemotactic transcriptional programs (Figure 3D). Combinations containing TGFB exhibited a large overlap of positively synergistic genes (EGF+TGFB = 32/110, OSM+TGFB = 32/65) (Figure 3E). These combinations also synergistically induced transcriptional programs related to ECM remodeling and cell motility, consistent with TGFB’s known role in promoting epithelial-to-mesenchymal transition and the emergent enlargement of cell cytoplasm in this condition.^46^ Notably, these EMT-related programs were not strongly induced by TGFB treatment alone and required either OSM or EGF in combination. This aligns with the observation from live-cell imaging that TGFB only induces motility and alterations in cell morphology in the presence of OSM or EGF (Figures 1D and 1E). Consistent with the live-cell image data, EGF+OSM and EGF+TGFB both downregulate epithelial differentiation processes (epithelium development, skin development). Epithelial cells that undergo a loss of epithelial identity, notably through epithelial-to-mesenchymal transition, exhibit increased motility and changes in cell morphology, which we observed in both treatment combinations.^47^ Negatively synergistic gene sets for EGF+OSM and EGF+TGFB had a large overlap, with 35 genes in common (Figure 3F), suggesting that these shared repressive transcriptional programs contribute to these processes (Figures 1D and 1E).

Given the critical role of cell-substrate interactions in regulating adhesion, migration, and morphodynamic changes, we examined whether integrin receptors or their extracellular matrix substrates were synergistically regulated in response to ligand combinations (Figure S3H). This analysis revealed that several integrin-related molecules exhibited significant synergy in expression. Notably, EGF+TGFB treatment synergistically upregulated collagen IV components and tenascin C, both of which are key ECM proteins involved in EMT.^48^^,^^49^ Additionally, both TGFB-containing combinations (EGF+TGFB and OSM+TGFB) synergistically upregulated integrin β3 (ITGB3), a well-established regulator and target of TGFB signaling.^50^ This suggests that either OSM or EGF may amplify a TGFB-driven feedforward loop through integrin β3 modulation. Given that integrin β3 is implicated in adhesion, migration, and breast cancer metastasis, particularly to the bone, its synergistic upregulation could have important functional consequences for the observed phenotypic changes in our study.^51^

### Partial least-squares regression uncovers transcriptional signatures driving cellular phenotype

We next sought to uncover molecular drivers of cellular phenotypes. Utilizing partial least-squares regression (PLSR), an efficient statistical prediction tool that is especially appropriate for small sample data with many possibly correlated variables,^30^ we constructed models to predict each of the four phenotypic metrics (Figure 1). The three biological replicates of imaged cells shown in Figure 1 were harvested for RNA sequencing, enabling direct linkage of image and RNA-seq data for each sample. This design leveraged biological variation in both phenotypic and transcriptional responses across replicates. Replicate-level phenotypic metrics and replicate-level Log Fold Change (LFC) gene expression compared to T0 control were used as PLSR inputs. We used leave-one-out analysis to determine the optimal number of latent variables and to evaluate model robustness.^52^ The Relative Root Mean Squared Error of Prediction (RRMSEP) for the four models ranged from 0.205 to 0.563, indicating a good fit.

**Figure 4:**
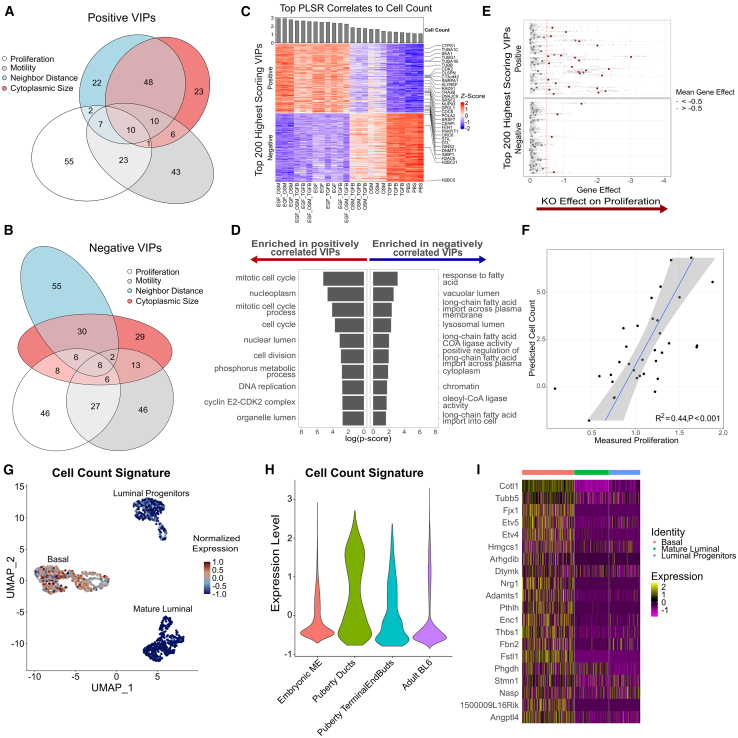
Partial least-squares regression (PLSR) links transcriptional signatures to phenotypic metrics (A-B) PLSR models were used to link RNA sequencing data to phenotypic metrics derived from live-cell imaging (cytoplasmic size, nearest neighbor distance, motility, and cell count). Overlap in top positive VIP genes for each PLSR model is visualized in (A), while overlap for top negative VIPs is shown in (B). (C) Z-scored gene expression of the top 100 positive VIPs and top 100 negative VIPs for the cell count PLSR model. (D) Gene set enrichment analysis of the top VIPs for the Cell Count model. (E) Validation of cell count PLSR model using the DEPMAP cancer dependency map (Project Achilles). Genes are shown in the same order as the top PLSR heatmap in (C). Genes positively correlated with Cell Count show significant enrichment for essentiality in 94 breast cancer cell lines (χ² = 902.41, p < 2.2e−16). Dots are labeled red for genes with mean gene effect scores >0.5 across all cell lines, indicating essentiality. Red vertical line indicates the threshold for determining gene essentiality. (F) Generalization of the Cell Count PLSR model to other cellular contexts. The model was applied to RNA-seq data from 34 breast cancer cell lines and showed significant correlation (R^2^ = 0.44, *p*-value <0.001) with experimental proliferation rates. Shaded region represents a 95% confidence interval. (G) UMAP representations of murine mammary ductal epithelial cells during puberty. Expression of the Cell Count PLSR gene signature is enriched in basal cells. (H) Violin plot shows cell count signature expression across three developmental stages—embryonic, puberty (including terminal end buds), and adult. Violin plot shows cell count signature expression across developmental stages. ME, mammary epithelial. BL6, C57BL/6 mouse strain. (I) Heatmap of basal mammary epithelial cell markers that overlap with Cell Count PLSR signature genes.

For each PLSR model, we identified the gene signature most strongly associated with phenotypic changes by calculating Variable Importance in Projection (VIP) scores.^53^ VIP scoring estimates the overall significance of each feature in the model without distinguishing between positively and negatively correlated features. For each model, we selected for further analysis the top 100 genes with the highest VIP scores and a positive correlation to the model’s first component and the top 100 genes with a negative correlation to the first component. There were varying degrees of overlap among the highest-scoring VIPs from different phenotypic signatures. The largest overlap was observed between the Nearest Neighbor Distance and Cytoplasmic Size signatures, likely due to the inverse relationship between cell spreading and cell clustering (Figures 4A and 4B). Similarly, there was overlap between the Cell Count and Motility signatures, which is expected as these biological processes are driven by similar molecular mechanisms.^54^ Despite these overlaps, a significant number of genes were uniquely identified as VIPs for each phenotype, indicating that the PLSR regression identifies distinct biological mechanisms. Gene set enrichment for the top negatively correlated VIPs for Nearest Neighbor Distance includes terms related to inflammatory response (acute-phase response, complement activation) (Figures S4A and S4B).^42^^,^^43^^,^^44^ The top positively correlated VIPs associated with cytoplasmic size are enriched in terms related to synthesis processes for cell membrane components (isoprenoid biosynthetic process, cholesterol biosynthetic process, phospholipid biosynthetic process) and cell-substrate interactions (cell-substrate junction, focal adhesion), indicating a shared transcriptional program involved in membrane and cytoskeletal remodeling that regulates cytoplasmic size (Figures S4C and S4D).

We next focused on exploring and validating the Cell Count PLSR model, which represents a well-studied phenotype that serves as an excellent test case of our approach to link image and molecular data. Genes positively correlated with cell count exhibited enrichment for gene sets canonically associated with proliferation, including mitotic cell cycle, DNA replication, and cyclin E2-CDK2 complex (Figures 4C and 4D). Among the positively correlated VIPs are essential components of mitosis, including several cell cycle checkpoint genes (CDK2, CDC6, CDCA4),^55^^,^^56^^,^^57^ microtubule regulation genes (TUBB, TUBB4B, and TUBG1),^58^ and growth-regulating secreted factors (AREG, IL1A, CSF3).^59^^,^^60^^,^^61^ Conversely, the top negatively correlated VIPs associated with cell count were enriched for cellular component terms such as vacuolar lumen and lysosomal lumen, implying an upregulation of autophagy components in the absence of proliferation, potentially due to cellular stress.^62^ These findings demonstrate that our PLSR-based approach can successfully capture known, biologically relevant pathways and processes associated with proliferation, validating our strategy to uncover molecular drivers of cellular phenotypes.

Assessing computational models using orthogonal approaches is crucial to ensure their robustness and reliability. Here, we leveraged independent, publicly available datasets to assess model generalizability and to validate our Cell Count model. First, we investigated whether the cell count signature genes are essential for viability across a panel of diverse cancer cell lines. We utilized the Project Achilles dataset from the Cancer Dependency Map Portal (DEPMAP), which used high-throughput CRISPR Cas9 knockouts to experimentally determine gene essentiality across thousands of cancer cell lines.^63^^,^^64^ The Gene Effect score measures the impact of gene knockout on cell viability. More negative scores indicate greater impact, with scores below −0.5 indicating cell depletion and below −1 indicating strong cell killing. A score of 0 means no effect on viability.

We examined the essentiality of the highest scoring VIPs included in our PLSR model by analyzing their experimentally determined Gene Effect scores for the 94 breast cancer cell lines included in the DEPMAP database (Figure 4E). VIP genes that showed the highest positive correlation with cell count were significantly more likely to have a Gene Effect score ≤ −0.5 as compared to genes with negatively correlated VIP scores (χ^2^ = 902.41, *p*-value <2.2e-16). Moreover, across all genes included in our Cell Count PLSR model, those identified as high-scoring VIPs were more frequently associated with a Gene Effect score ≤ −0.5 than were genes with low-scoring VIPs (χ^2^ = 3841.6, *p*-value <2.2e-16), demonstrating the predictive capability of our model in identifying genes causally related to cell cycle and viability in a diverse panel of breast cancer cell lines (Figure S4E). Notably, genes with both high VIP scores and large Gene Effect scores include canonical cell cycle components (CDC20, PLK1, GINS1)^58^ as well as recently discovered regulators (ALYREF)^65^ and prognostic markers of breast carcinogenesis (GINS2).^66^

We also assessed the generalizability of the cell count PLSR model to predict proliferation in other cell line models. We analyzed publicly available datasets comprised of paired RNA-seq and doubling time measurements from 34 breast cancer cell lines.^67^ For each cell line, we input the log2-normalized gene expression data into our model to predict cell count. The model’s predicted cell count was significantly correlated with experimental doubling time measurements (R^2^ = 0.44, *p*-value <0.001), further supporting the generalizability of the model (Figure 4F). Together, this underscores the power of perturbing a single experimental system to gain insights into mechanisms governing cell viability operable across diverse biological contexts.

To assess the biological relevance of our PLSR-derived gene signatures *in vivo*, we analyzed publicly available single-cell RNA sequencing data of murine mammary gland ductal epithelial cells across embryonic, pubertal, and adult stages.^68^ As expected, ductal epithelium segregated into three distinct subpopulations based on gene expression: basal, mature luminal, and luminal progenitors. We examined the expression of our PLSR signatures in these subpopulations and found that basal cells predominantly expressed our Cell Count signature, followed by luminal progenitors, and finally mature luminal cells (Figures 4G and S4F). This aligns with the known proliferative capacities of these subpopulations.^69^ Conversely, our Motility signature was almost exclusively expressed by mature luminal cells, supporting the “go-or-grow” model of cellular behavior, wherein cells with lower proliferative capacity exhibit higher migratory potential (Figures S4G and S4H).^70^

Next, we analyzed the expression of the Cell Count signature across developmental stages and observed its upregulation during puberty, when progenitor epithelial cell population peaks (Figure 4H).^71^ Further analysis of genes within the Cell Count signature that were upregulated in basal cells revealed enrichment for genes linked to stemness in mammary tissues, including STMN1, ETV4, and ETV5 (Figure 4I).^72^^,^^73^ These findings provide additional biological validation for our PLSR-derived gene signatures and support their relevance in identifying key molecular drivers of proliferation and motility in epithelial cell populations *in vivo*. These results show that the Cell Count model extends beyond MCF10A to diverse cellular contexts and provides a means to directly connect molecular and image-based measurements. Together, the findings highlight the robustness of this approach in uncovering molecular programs that drive complex phenotypic responses.

### Synergistic transcriptional upregulation of CXCR2 chemotactic signaling molecules via CREB activation promotes increased cell motility

**Figure 5:**
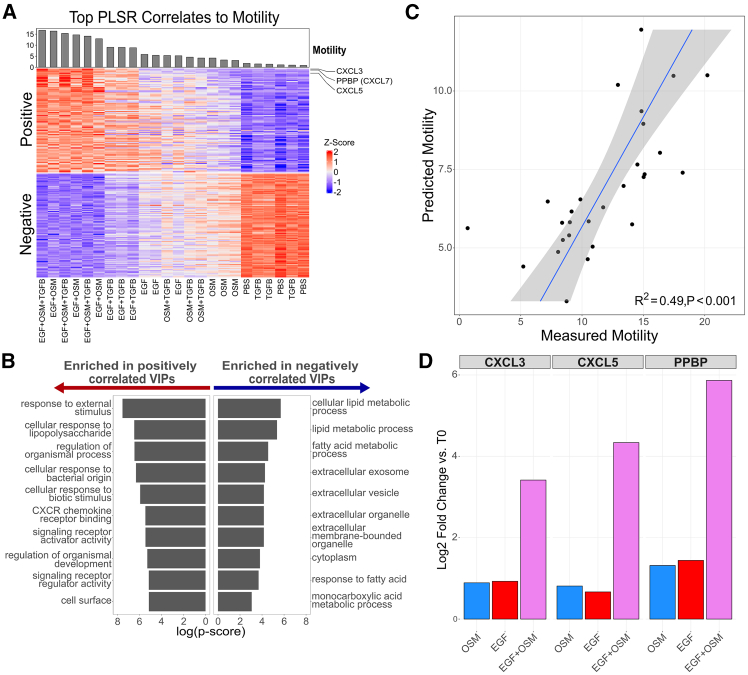
Expression of CXCR2 Agonists Correlates with Cell Motility (A) Z-scored expression for the top 100 VIP genes positively and negatively correlated with cell motility. (B) Gene set enrichment analysis of top VIPs, showing significant enrichment in pathways related to CXCR chemokine receptor binding. (C) Correlation between predicted cell motility values from the Cell Motility PLSR model and experimentally measured migration rates from 28 breast cancer cell lines. A significant correlation (R^2^ = 0.49, *p*-value <0.001) between predicted motility and experimental migration rates ports the relevance of the model in capturing motility-associated biological pathways. Shaded region represents 95% confidence interval. (D) LFC values compared to T0 control for the EGF+OSM combination condition and respective single-ligand conditions show the synergistic upregulation of CXCR2 agonists (CXCL3, CXCL5, and PPBP).

To investigate the molecular mechanisms driving cell motility, we analyzed our Cell Motility model and the associated gene signature (Figure 5A). Gene set enrichment analysis revealed that genes positively correlated with cell motility are significantly enriched in pathways related to responses to external and biotic stimuli, signaling receptor activator activity, and CXCR chemokine receptor binding (Figure 5B). To assess model generalizability, we leveraged our Cell Motility PLSR model to predict cell motility from publicly available RNA-seq profiles from 28 breast cancer cell lines and compared this to experimentally measured migration rates.^54^^,^^67^ The predicted cell motility values were strongly correlated with experimentally measured migration rates, indicating that our model captures motility-related molecular programs operable across diverse cell contexts (R^2^ = 0.49, *p*-value <0.001) (Figure 5C).

Having established the validity of our Cell Motility model, we next explored it to uncover biological mechanisms driving this phenotypic response. The topmost important genes include CXCL3, PPBP (CXCL7), and CXCL5, ranking first, second, and fifth in VIP scores, respectively (Figure 5D). These genes encode chemotactic ligands that signal through the CXCR2 chemokine receptor, a pathway known to enhance mammary epithelial cell migration.^74^ Furthermore, our previous RNA-seq analysis showed that CXCL3, CXCL5, and PPBP are synergistically upregulated under the EGF+OSM condition, which produced the most significant increase in cell motility among all conditions tested (Figures 1D and 5D). Motivated by this, we focused on the EGF+OSM combination condition to functionally investigate the mechanism by which this combined treatment synergistically enhances motility.

**Figure 6:**
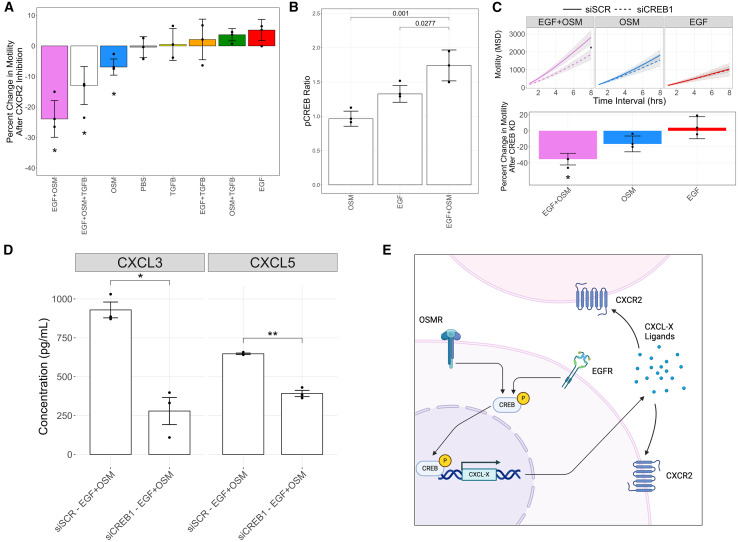
Synergistic transcriptional upregulation of CXCR2 chemotactic signaling molecules via CREB activation promotes increased cell motility (A) Cell motility assays following treatment with single and combination ligand panels in the presence or absence of AZD5069, a CXCR2 inhibitor. CXCR2 inhibition significantly decreased cell motility in the EGF+OSM, EGF, and OSM conditions. Data shown as median change in motility and error bars representing the standard deviation from three biological replicates. ANOVA followed by post-hoc Tukey’s honest significant difference test was used to assess significance, with *p*-value <0.05 considered significant (*n* = 3). ∗*p* < 0.05. (B) Reverse phase protein array (RPPA) analysis 1 h after treatment with EGF, OSM, and EGF+OSM. Median phosphorylation ratio is shown, with error bars representing standard deviation. Statistically significant changes in protein expression (*p*-value <0.05) were assessed using Dunnett’s test (*n* = 3). (C) Effects of CREB knockdown on the cell motility of cells treated with EGF, OSM, or EGF+OSM. Mean squared displacement (MSD) is shown in the top panel and change in motility is shown in the bottom. Median change in motility is shown, with error bars representing standard deviation. Tukey’s honest significant difference test was used to assess significance with *p*-value <0.05 considered significant (*n* = 3). ∗*p* < 0.05. (D) ELISA analysis of chemokine expression in CREB knockdown cells following EGF+OSM treatment. Bar plots depict mean protein expression in conditioned media, with error bars representing the 95% confidence interval. Student’s t test was used to assess significance (*p*-value <0.05) (*n* = 3). ∗*p* < 0.05, ∗∗*p* < 0.01. (E) Putative mechanism of the synergistic activation of CREB in response to combined EGF and OSM stimulation drives the upregulation of CXCL3, CXCL5, and PPBP, leading to increased cell motility via CXCR2 activation.

We hypothesized that the upregulation of CXCL3, CXCL5, and PPBP (CXCL7) contributes to the increased cell motility observed in the EGF+OSM condition compared to EGF and OSM single-ligand conditions. To test this hypothesis, we first asked whether ativation of CXCR2–the common receptor for CXCL3, CXCL5, and PPBP–influences cell motility in the EGF+OSM condition. We treated MCF10A cells with the ligand panel in the presence of AZD5069, a small molecule inhibitor of CXCR2 receptor activation, and then assessed cell motility. CXCR2 inhibition significantly suppressed cell motility in the EGF+OSM+TGFB, EGF+OSM, and OSM conditions, with the most substantial decrease observed in the EGF+OSM condition (23.9% median decrease across three biological replicates) (Figures 6Aand S5A). To test whether CXCR2 chemokine signaling is sufficient to promote motility, we treated MCF10A cells with exogenous CXCL3 or CXCL5 in the presence of PBS, EGF, OSM, or EGF+OSM and measured cell motility. We found that in all conditions, the addition of either chemokine led to a significant increase in motility, with the most pronounced effects observed in the PBS and OSM conditions (Figure S5B). These results indicate that CXCL3 and CXCL5 signaling can promote cell motility independent of ligand co-treatment and suggest that MCF10A cells are primed to respond to these chemokines.

We next sought to identify the transcriptional regulators driving the synergistic upregulation of CXCL3, CXCL5, and PPBP in the EGF+OSM condition, hypothesizing that their activation would follow a similar synergistic pattern. To identify transcriptional regulators synergistically activated by EGF+OSM, we assessed proteomic changes with Reverse Phase Protein Array (RPPA) analysis 1 h after ligand treatment (Table S2).^75^ We identified 3 proteins with a statistically significant change (*p*-value <0.05) in expression between the EGF+OSM condition as compared to both single ligand conditions: P70 S6 Kinase (pThr389), S6 (pSer240/244), and CREB (pS133) (Figures 6C and S5C). CREB is a transcription factor known to enhance CXCL3, CXCL5, and PPBP expression,^76^ and its upregulation is consistent with our hypothesis that the enhancement of chemotactic signaling contributes to increased motility after EGF+OSM treatment.

We functionally validated CREB’s role in motility by performing siRNA knock-down in the presence of EGF, OSM, and EGF+OSM treatments, followed by the assessment of cell motility. CREB inhibition had minimal impact on EGF treated cells, a modest impact on OSM treated cells, and the greatest impact on EGF+OSM treated cells, mirroring the effects of CXCR2 inhibition (Figure 6D). The knockdown of CREB was confirmed through qPCR (Figure S5D). To assess the relationship between CREB and chemokine expression, we measured CXCL3, CXCL5, and PPBP levels after EGF+OSM treatment with or without CREB knockdown (Tables S3 and S4). CREB knockdown reduced CXCL3 and CXCL5 expression, suggesting direct transcriptional regulation (Figure S5E). ELISA of conditioned media further confirmed this effect at the protein level, with significantly lower CXCL3 and CXCL5 secretion following CREB knockdown, supporting a role for CREB in driving chemokine production in response to EGF+OSM (Figure 6D and Table S5). In summary, our findings show that the synergistic activation of CREB in response to combined EGF and OSM signaling drives the upregulation of the chemokines CXCL3, CXCL5, and PPBP, promoting increased cell motility (Figure 6E).

## Discussion

Cells operate within complex microenvironments in which signals from the local environment are continuously changing. Cells must integrate these signals at the molecular level to yield coordinated changes in cellular function. Most prior studies of treatment combinations have focused on therapeutic inhibitors and a limited number of phenotypes, primarily cell viability and proliferation. In contrast, this study sought to expand these concepts to ligand treatments and a richer set of phenotypic responses. By systematically analyzing the effects of EGF, OSM, and TGFB—three ligands that engage distinct molecular and cellular programs—we demonstrate that combinatorial ligand treatments can elicit emergent phenotypic behaviors not predictable from single-ligand responses, including changes in cell proliferation, motility, morphology, and spatial organization. These results demonstrate that the combined effects of ligand treatments can exceed the sum of their individual responses, emphasizing the challenge of predicting phenotypic responses from single-ligand treatment data.

Gene expression analysis revealed that combination treatments largely reflect the transcriptional profiles of their respective single-ligand conditions, suggesting an additive model of regulation. However, despite this general trend, some combinations also activated distinct transcriptional programs not apparent in single-ligand treatments. Notably, gene programs associated with epithelial differentiation were downregulated across nearly all treatment conditions, apart from TGFB. Given TGFB’s well-established role in epithelial-to-mesenchymal transition (EMT), this result suggests that TGFB alone may be insufficient to drive EMT in MCF10A cells and that EGF signaling may be required to fully activate this transition,^4^^,^^71^^,^^72^ demonstrating that well-studied ligand-induced phenotypes may depend on the presence of other ligands in the microenvironment.

While broad transcriptional changes in combination treatments were largely predictable from additive models, transcription factor (TF) enrichment analysis uncovered uniquely activated regulatory programs. Specifically, we identified 28 transcriptional regulators enriched only in combination treatments, including TFs involved in epithelial differentiation, immune signaling, and growth factor response. This suggests that while many transcriptional responses align with an additive framework, certain regulatory mechanisms are selectively engaged only when multiple signaling pathways converge. This activation of unique transcriptional programs in combination conditions may drive the distinct phenotypic effects observed, despite the additive nature of the overall transcriptional landscape. These findings align with prior studies showing that small degrees of transcriptional synergy induce large phenotypic changes.^24^

Building on prior research,^16^ we utilized an HSA-based modeling approach to define synergistic transcriptional programs.^34^ This approach uncovered subtle but significant synergistic gene sets specific to each ligand combination. While most studies have focused on phenotypic synergy, extending these frameworks to molecular synergy provides valuable insights into the underlying mechanisms driving combined ligand effects. Our studies support the adoption of existing frameworks designed for drug-induced changes in viability to gain insights into complex phenotypic responses. An important consideration in interpreting these results is the heterogeneity of cellular responses. Single-cell motility analyses revealed that conditions containing both EGF and OSM exhibited a bimodal motility distribution, suggesting the presence of distinct subpopulations with different migratory behaviors. Bulk transcriptional analyses may obscure these subpopulation-specific transcriptional programs. Future work incorporating spatial^77^ and single-cell transcriptomic approaches^78^ could help disentangle the molecular networks responsible for these heterogeneous responses.

The use of partial least-squares regression (PLSR) to connect transcriptional data with phenotypic outcomes is a significant strength of this study. Identifying gene signatures linked to cell count, motility, spatial organization, and cytoplasmic size offers insights into the molecular drivers of these processes. The validation of the PLSR model with publicly available datasets, including the Cancer Dependency Map, underscores the robustness and generalizability of our PLSR models, with potential applications for identifying critical regulators of cell proliferation and survival.^54^^,^^63^^,^^64^^,^^67^ Furthermore, this approach holds significant promise for uncovering regulators of cell phenotype by linking previously uncharacterized genes to specific cellular behaviors.

Our live-cell studies revealed complex temporal dynamics as phenotypic responses emerged after perturbation. For example, changes in cell number showed condition specific dynamics, suggesting that deeper analysis of changes in cell cycle phase over time could further elucidate mechanisms driving response. In future work, expanding these frameworks to explicitly model the dynamics of phenotypic change will be valuable, for example, using fluorescent cell cycle reporters and trajectory embedding to track temporal responses.^79^^,^^80^^,^^81^^,^^82^^,^^83^ These techniques enable the dissection of heterogeneous and dynamic phenotypic transitions at single-cell resolution, which cannot be captured by endpoint measurements alone. Incorporating similar time-resolved and reporter-based strategies within the context of combinatorial ligand perturbations could reveal how the timing, duration, and sequence of signaling events contribute to emergent phenotypic outcomes such as proliferation, migration, and differentiation. Alternative machine learning approaches, such as random forests or neural networks, could also be employed to capture more complex non-linear relationships between transcriptional data and phenotypic outcomes.^84^^,^^85^^,^^86^^,^^87^ These methods may offer complementary insights and improve our understanding of the complex signaling networks governing cellular behavior.

Our analysis of CXCR2 chemotactic signaling and CREB activation provides important mechanistic insights into molecular drivers of cell motility. The synergistic upregulation of CXCL3, CXCL5, and PPBP in the EGF+OSM condition, along with the effects of CXCR2 inhibition and CREB knockdown, highlights a key signaling axis driving cell motility. CREB’s role in promoting chemokine expression further clarifies the molecular mechanisms underlying the observed changes. We demonstrated that EGF+OSM, only when applied in combination, phosphorylates and activates CREB transcription factor, leading to the transcriptional upregulation of CXCR2 receptor agonists. The activation of CXCR2 then contributes to increased cell motility.

Previous studies have established CREB’s role in promoting chemokine activity in non-small cell lung cancer cells, myeloid cells, and erythroblasts.^76^^,^^88^^,^^89^ While EGF is known to activate CREB in various contexts, including in breast tissue,^90^ our studies indicate that in MCF10A cells, this pathway is uniquely activated by the combined presence of OSM and EGF. Additionally, prior research has shown that CXCR2 is overexpressed in breast cancer epithelial cells and enhances malignant cell migration.^91^^,^^92^ Here, we provide evidence for an autocrine signaling mechanism achieving similar outcomes for cell motility. However, it is important to note that the observed phenotypic changes likely also involve protein and signaling-driven mechanisms. While this study focused on the CREB-CXCR2 axis, future research could investigate other interacting pathways to provide a more comprehensive view of the regulatory networks driving motility. For example, pinpointing which CXCR2 agonists actively bind the receptor or investigating other transcription factors or co-regulators involved in chemokine expression and motility could provide deeper insights.

The ability to manipulate microenvironmental signaling through ligand or drug combinations has important therapeutic implications. Clinically, targeted therapies such as monoclonal antibodies, kinase inhibitors, and cytokine therapies allow for some degree of control of microenvironmental signaling in diseases such as cancer.^93^^,^^94^^,^^95^ Key to translating these concepts to the clinic is modeling and understanding how combinations of signals influence disease progression. Theoretical and computational models, including machine learning approaches, can aid in predicting how multiple ligands interact, offering insights into how many signals can be effectively manipulated to control cellular responses and therapeutic outcomes.^84^^,^^87^^,^^96^

Overall, this study offers a comprehensive analysis of how EGF, OSM, and TGFB signaling pathways interact to influence cellular responses through complex transcriptional programs. The integration of transcriptomic and phenotypic data using machine learning approaches enhances our understanding of the molecular mechanisms governing cell behavior, with potential implications for developing targeted therapeutic strategies to modulate cell motility and proliferation in cancer and other diseases.

### Limitations of the study

This study has several limitations that should be addressed in future research. First, our study relied on endpoint measurements to quantify proliferation, which does not capture the dynamic temporal changes in cell number observed at earlier timepoints. This simplification may obscure biologically relevant differences in proliferative responses among ligand conditions. Future work incorporating live-cell imaging with cell cycle reporters^80^^,^^81^ will be essential to dissect the heterogeneous and dynamic nature of proliferative signaling. Additionally, while we focused on specific quantitative changes in cellular phenotype, many other aspects remain unexplored, such as metabolic activity, apoptosis, and differentiation status. Examining these phenotypes could provide critical insights into how ligand combinations influence cellular behavior. For instance, changes in metabolic activity could elucidate the energetic requirements for motility^97^; apoptosis assays might reveal how ligand signaling impacts cell survival^98^; and the assessment of differentiation markers could help determine whether ligand combinations push cells toward specific differentiation states.^99^ Additionally, investigating chromatin remodeling and transcriptional dynamics could uncover upstream regulatory mechanisms driving the observed phenotypic changes.^100^

Second, our study used a single, saturating dose for each ligand, chosen based on previous dose-response experiments demonstrating that higher concentrations did not further increase proliferation.^11^ However, we did not explore dose dependence for other phenotypes, such as motility, where ligand interactions may exhibit non-linear or threshold-dependent effects. Future studies incorporating a range of ligand concentrations could explore the amplification of responses at lower doses. Additionally, our approach involved culturing cells in growth factor-depleted conditions to isolate ligand-specific effects. While this allowed us to establish a controlled baseline and minimize confounding signals, it does not fully recapitulate the dynamic signaling environment *in vivo*, where cells are continuously exposed to extracellular cues. Future studies could assess how ligand synergy operates in more physiologically relevant contexts, such as in serum-rich conditions or co-culture systems with stromal or immune cells.

Third, our experiments focused on the MCF10A mammary epithelial cell model. While we validated our results with external cancer cell line datasets containing thousands of paired RNA-seq and phenotypic profiles,^64^ as well as single-cell RNA-seq from murine mammary epithelial cells during development,^68^ future studies could expand the types of cells studied, including primary cells that more closely mimic *in vivo* physiological states.^101^ The mechanisms we identified in this study are likely also context-dependent, as different cell types—stromal and immune—may exhibit distinct responses to microenvironmental signals due to variations in receptor expression, intracellular signaling networks, and epigenetic landscapes. For example, TGFB promotes epithelial-to-mesenchymal transition (EMT) in epithelial cells, driving motility and morphological changes, whereas in immune cells, TGFB plays an immunosuppressive role by inhibiting T cell activation and promoting regulatory T cell differentiation.^102^ Similarly, we have shown that OSM enhances motility and proliferation in epithelial cells by activating the JAK-STAT pathway, while in muscle cells, OSM treatment leads to growth arrest.^11^^,^^103^ To determine the extent to which our findings generalize to other cell types, future studies could employ large-scale screening approaches, such as high-content imaging or single-cell transcriptomics, across diverse cellular contexts. A systematic evaluation of ligand interactions in primary epithelial cells, fibroblasts, and immune cells would help elucidate the broader applicability of our findings and provide insights into how microenvironmental signaling networks drive cell type-specific responses. Additionally, using organoids, a more complex model system that includes additional cell-cell contacts and extracellular matrix, could help determine the generalizability of our findings across different biological contexts.^104^

Lastly, while our study observed various types of cell motility, we did not differentiate between distinct motility behaviors. Understanding the differences between random motility, directed migration, and collective movement could offer deeper insights into the regulation of these processes.^105^ To address this, future experiments could include chemotactic gradient assays to evaluate directed migration,^106^ scratch assays to study wound healing-like behavior,^107^ and 3D model systems to investigate the contributions of the ECM.^108^ Additionally, future work should explore how ligand synergy is modulated by substrate mechanics, which could further clarify how matrix adhesion impacts cellular responses in different microenvironmental contexts. Such approaches would provide a more nuanced understanding of how specific signaling pathways influence distinct motility types.

## Resource availability

### Lead contact

Further information and requests should be directed to the lead contact, Laura M Heiser (heiserl@ohsu.edu).

### Materials availability

This study did not generate new unique reagents.

### Data and code availability

• Bulk RNA sequence data have been deposited at Gene Expression Omnibus: PRJNA1189550 and are publicly available as of the date of publication. Accession numbers are listed in the key resources table.
• Live-cell imaging data have been deposited at Zenodo: https://doi.org/10.5281/zenodo.14261795 and are publicly available as of the date of publication.
• LC-MS/MS data have been deposited at ProteomeXchange: PXD067098 and are publicly available as of the date of publication. Accession numbers are listed in the key resources table.
• All original code has been deposited at Github and is publicly available as of the date of publication at: https://github.com/mcleania/MCF10A_LigandCombination.
• Any additional information required to reanalyze the data reported in this article is available from the lead contact upon request.

## Acknowledgments

This work was supported by NIH research grants U54-CA209988, U54 HG008100, and the 10.13039/100030442Anna Fuller Fund (L.M.H.) and 10.13039/100000054NCI 5U01CA243004 (A.E.). OHSU Massively Parallel Sequencing Shared Resource receives support from the OHSU Knight Cancer Institute NCI Cancer Center Support Grant P30CA069533. The authors acknowledge Lauren Kronebusch for help with article editing. Cartoons created with Biorender.com.

## Author contributions

Conceptualization: LMH, ICM, SMG, and AE; investigation: ICM, SMG, IP; methodology: LMH and ICM; formal analysis: ICM, DSD, and JC; visualization: ICM; supervision: LMH; writing—original draft: ICM, writing—review and editing: LMH, ICM, DSD, JC, IP, and AE.

## Declaration of interests

AE is a founder of the startup Prisma Therapeutics and a member of its scientific advisory board.

## STAR★Methods

### Key resources table

**Table:** 

REAGENT or RESOURCE	SOURCE	IDENTIFIER
**Chemicals, peptides, and recombinant proteins**		

Recombinant Human EGF Protein, CF	R&D Systems	Cat#236-EG; UniProtKB:P01133
Recombinant Human Oncostatin M (HEK-293-expressed) Protein	R&D Systems	Cat#8475-OM; UniProtKB:P13725
Recombinant Human TGF-beta 1 Protein	R&D Systems	Cat#240-B; UniProtKB:P01137
Human GRO-gamma (CXCL3) Recombinant Protein, PeproTech® - 10ug	ThermoFisher Scientific	Cat#300-40-10UG; UniProtKB:P19876
Human ENA-78 (CXCL5) (5-78 aa) Recombinant Protein, PeproTech®	ThermoFisher Scientific	Cat#300-22-5UG; UniProtKB:P42830
AZD-5069	MedChemExpress	Cat#HY-19855; PubChemCID:56645576
Trametinib (GSK1120212)	SelleckChem	Cat#S2673; PubChemCID:11707110
Alpelisib (BYL719)	SelleckChem	Cat#S2814; PubChemCID:56649450

**Critical commercial assays**		

RNeasy Mini Kit	Qiagen	Cat#74104
TruSeq® Stranded mRNA Library Prep (48 Samples)	Illumina	Cat#20020594
iScript cDNA Synthesis Kit	BioRad	Cat#1708891
TMTpro 18-plex, TMTpro-134C, and TMTpro-135N Label Reagents	Thermo Fisher Scientific	Cat#A52045
Human C-X-C motif chemokine 3 (CXCL3) Elisa Kit	AFG Scientific	Cat#EK712056
Human Epithelial neutrophil activating peptide 78 (ENA-78,CXCL5) Elisa Kit	AFG Scientific	Cat#EK712055

**Deposited data**		

BCCL RNAseq Data	Heiser et al.^67^	https://doi.org/10.1073/pnas.1018854108
BCCL Motility Quantification	Nair et al.^54^	https://doi.org/10.1038/s41598-019-47440-w
DEPMAP Achilles Data	Tsherniak et al.^64^	https://doi.org/10.1016/j.cell.2017.06.010
RNAseq data from combination ligand treatments	This paper	GEO: GSE282654
Live-cell imaging from combination ligand treatments	This paper	Zenodo: https://doi.org/10.5281/zenodo.14261795
LC-MS/MS from single ligand treatments	This paper	ProteomeXchange: PXD067098
ScRNA-seq profiling of mouse mammary epithelial cells	Pal et al.^68^	GEO: GSE103275

**Experimental models: Cell lines**		

Human: MCF10A line	Gift from Gordon Mills (OHSU)	RRID:CVCL_0598

**Oligonucleotides**		

ON-TARGETplus siRNA: siCREB1	Horizon Discovery	Cat#L-003619-00-0005
ON-TARGETplus siRNA: non-targeting	Horizon Discovery	Cat#L-003619-00-0005
ON-TARGETplus siRNA: siCREB1	Horizon Discovery	Cat#L-003619-00-0005
ON-TARGETplus siRNA: non-targeting	Horizon Discovery	Cat#L-003619-00-0005

**Software and algorithms**		

Fiji	Schindelin et al.^109^	https://imagej.net/software/fiji/
CellPose 2.0	Pachitariu et al.^110^	https://www.cellpose.org/
Baxtor Algorithm	Magnusson et al.^111^	https://github.com/klasma/BaxterAlgorithms
RStudio v. 3.5.0	NA	https://posit.co/products/open-source/rstudio/
TrimGalore v. 0.4.3	Martin.^112^	https://zenodo.org/records/7598955
CutAdapt v. 1.10	Martin.^112^	https://github.com/FelixKrueger/TrimGalore/tree/0.6.10
FastQC v. 0.11.5	NA	http://www.bioinformatics.babraham.ac.uk/projects/fastqc/
Kallisto v. 0.46.2	Bray et al.^113^	https://pachterlab.github.io/kallisto/
tximport v. 1.8.0	Soneson et al.^114^	https://www.bioconductor.org/packages/release/bioc/html/tximport
DESeq2 v. 1.24.0	Love et al.^115^	https://bioconductor.org/packages/release/bioc/html/DESeq2
Priori	Yashar et al.^39^	https://github.com/ohsu-comp-bio/regulon-enrichment
GProfiler	Kolberg et al.^42^	https://cran.r-project.org/web/packages/gprofiler2/
Seurat v. 3.0	Stuart et al.^116^	https://cran.r-project.org/web/packages/Seurat/
PLS	Mevik et al.^117^	https://cran.r-project.org/web/packages/pls/
FragPipe v 18.0	Yu et al.^118^	https://fragpipe.nesvilab.org/
Original code	This study	https://github.com/mcleania/MCF10A_LigandCombination

### Experimental model and study participant details

#### Cell lines

MCF10A (female) cells were a gift from Gordon Mills (OHSU). Cell identity was confirmed by short tandem repeat (STR) profiling and cells were routinely tested for mycoplasma. Cells were minimally passaged prior to experiments and sample collection. For growth and passage, cells were cultured at 37°C in growth media containing DMEM/F12 (Invitrogen #11330-032), 5% horse serum (Sigma #H1138), 20 ng/ml EGF (R&D Systems #236-EG), 0.5 μg/ml hydrocortisone (Sigma #H-4001), 100 ng/ml cholera toxin (Sigma #C8052), 10 μg/ml insulin (Sigma #I9278), and 1% Pen/Strep (Invitrogen #15070-063). For perturbation experiments, assay media was used, composed of DMEM/F12, 5% horse serum, 0.5 μg/ml hydrocortisone, 100 ng/ml cholera toxin, and 1% Pen/Strep.

### Method details

#### Ligand perturbation experiments

MCF10A cells were grown to 50-80% confluence in GM and detached using 0.05% trypsin-EDTA (Thermo Fisher Scientific #25300-054). Post-detachment, 5,000 cells were seeded into collagen-1 (Cultrex #3442-050-01) coated 24-well plates (Thermo Fisher Scientific #267062) in growth media. Six hours after seeding, cells were washed with PBS and assay media was added. After an 18-hour incubation in the new media, cells were treated with single ligand or combinations of ligands in fresh growth factor-free media: 10 ng/ml EGF (1.56 nM) (R&D Systems #236-EG), 10 ng/ml OSM (455 pM) (R&D Systems #8475-OM), 10 ng/ml TGFB (500 pM) (R&D Systems #240-B), 5ng/mL CXCL3 (6.32nM) (ThermoFisher Scientific # 300-40), and 5ng/mL CXCL5 (4.17 nM) (ThermoFisher Scientific # 300-40). We selected a concentration of 10 ng/mL for EGF, OSM and TGFB based on our previous dose-response studies, which demonstrated that this dose is saturating for proliferation and elicits robust signaling responses without further increases at higher concentrations.^11^

#### Live-cell imaging and image analysis

Live-cell imaging was performed using the Incucyte S3 microscope (Essen BioScience, #4647). Images were captured every 30 minutes for up to 48 hours. Live-cell image stacks were then registered using a custom Fiji script^109^ and segmented with CellPose.^110^ Image tracking was carried out using the Baxter Algorithms pipeline.^111^

All analysis of cell tracking data was performed in RStudio.^119^ The cell count metric was determined by counting the number of cells per field and normalizing this count by the T0 count for that field. Nearest neighbor distances were measured by calculating the pixel Euclidean distances from each cell centroid to the centroid of the second nearest cell in the imaging field. To account for variations in cell count, the mean nearest neighbor distances for each image were normalized by the expected mean distance to the nearest neighboring cell if the cells were distributed randomly.^120^ Cytoplasmic size was calculated as the average cytoplasmic size 24 hours after ligand addition. Cell motility was quantified by first removing tracks with distance jumps greater than 200 pixels in 30 minutes. Motility was estimated as the slope of the mean squared displacement (MSD)^121^ over time intervals ranging from 30 minutes to 6 hours. The slope of the MSD for each treatment was derived by constructing a linear model comparing MSD to the time interval. This value is proportional to the diffusion coefficient for Brownian motion.^121^

#### Liquid chromatography with tandem mass spectrometry

Cells from ligand treated MCF10A cells were washed with cold PBS twice, then collected by scraping with a cell scraper then immediately snap-frozen in liquid nitrogen. Protein extracts were prepared using the GuHCl method as previously described.^122^ Samples (30 μg each) were digested overnight at 37°C with sequencing-grade trypsin (1:50 enzyme-to-protein ratio), labeled using TMTpro 18-plex reagents (Thermo Fisher Scientific), and quenched with 5% hydroxylamine. Labeled peptides were pooled, desalted using C18 cartridges, dried, and fractionated by high-pH reverse-phase chromatography on an XBridge BEH C18 column into 96 fractions, which were concatenated into 24 for total proteome analysis. Five percent of each fraction was reserved for proteomic analysis, and the remainder concatenated into 12 fractions for phosphopeptide enrichment using Fe-NTA magnetic beads. LC-MS/MS analysis was performed on an Exploris 480 mass spectrometer coupled to a NEO Vanquish UHPLC system, with peptides separated over a 120-min gradient on a PepMap RSLC C18 column. MS1 scans were acquired at 120,000 resolution and MS2 in DDA mode at 45,000 resolution with dynamic exclusion (30 s). Raw data were processed in FragPipe (v18.0)^118^ with MSFragger against the UniProt Homo sapiens database (December 2024), allowing two missed cleavages, TMTpro fixed modifications (K, N-term), variable methionine oxidation and phosphorylation (S/T/Y), and mass tolerances of 20 ppm (precursor) and 0.05 Da (fragment). Peptide-spectrum matches were filtered at 1% FDR using Philosopher, and reporter ion intensities normalized and quantified with IonQuant. The full dataset of mass to charge ratios are reported in Table S1.

#### RNAseq generation and analysis

MCF10A cells were transferred into RLT Plus buffer (Qiagen) containing 1% β-ME, flash-frozen in liquid nitrogen, and stored at −80°C until RNA extraction. Total RNA was isolated from the frozen samples using the Qiagen RNeasy Mini Kit. cDNA libraries were prepared from poly(A)-selected RNA using the Illumina TruSeq Stranded mRNA Library Preparation kit. The Illumina HiSeq 2500 platform to generate 100-bp paired-end reads. Short read sequencing assays were performed by the OHSU Massively Parallel Sequencing Shared Resource.

RNAseq data was preprocessed and aligned using a pipeline adapted from Tatlow et al.^112^ TrimGalore (v. 0.4.3) was used to trim adapter sequences and low-quality bases using CutAdapt (v. 1.10)^113^ and to generate FASTQ quality reports using FastQC (v. 0.11.5). After adapter trimming, reads were filtered to have a minimum length of 30 bp. Trimmed reads were pseudo-aligned to the GENCODE V24 transcriptome (GRCh38.p5) using Kallisto (v. 0.46.2).^114^ Gene-level quantifications were obtained from transcript-level abundance estimates using the R package tximport (v. 1.8.0) in R (v. 3.5.0).^115^

We performed multiple differential gene expression analyses, comparing each ligand condition to time zero controls and comparing each two-ligand combination condition to the respective single ligand conditions. Both sets of analyses were conducted using RNA-seq gene-level summaries with the R package DESeq2 (version 1.24.0).^123^

Transcription factor enrichment scores were calculated using Priori with default settings, using TPM values as input.^39^ Gene modules were identified through K-means clustering of the top 200 most up-regulated and top 200 most down-regulated genes, employing the ComplexHeatmap package.^116^ The number of clusters was determined by gap analysis.

Gene set enrichment analyses were conducted using the Gprofiler package, focusing on Gene Ontology categories for Biological Process, Molecular Function, and Cellular Component.^42^^,^^43^^,^^44^ Gene sets were considered statistically significant if the adjusted *p*-value was below 0.01. The most enriched gene sets for each analysis were selected for visualization by ranking the gene sets first by the smallest *p*-value and subsequently by the highest odds ratio. *p*-value adjustments for multiple comparisons were made using the g:SCS method from the Gprofiler package.^42^

#### Partial least squares regression and validation

PLSR models were built using replicate-level gene expression data as the independent variable to predict the replicate phenotypic metrics as the dependent variable. This pairing of replicates allowed us to utilize biological variation in both phenotypic and transcriptional responses. To exclude low-variance genes from the model, we used the VST method in the Seurat RStudio package (Version 3) to select the top 2,500 most variable genes for input.^117^ Gene expression for each condition was normalized to T0 and scaled. Replicate level phenotypic metrics used as input into the models were derived from live-cell imaging experiments, as described in the live-cell imaging and image analysis section. Specifically, the model included the following features: T0-normalized cell count at 24 hours; nearest neighbor distance at 24 hours, normalized to the expected mean distance if cells were randomly distributed; cytoplasmic size at 24 hours; and motility, quantified as the slope of the mean squared displacement (MSD) over time intervals ranging from 30 minutes to 6 hours. All phenotypic metrics were scaled prior to model training. The PLSR models were constructed using the PLS package in RStudio.^124^ The number of components in each model was determined by identifying the elbows in the relative root mean squared error plots. Leave-one-out analysis was conducted to assess robustness. Genes were ranked by importance in the model by calculating the Variable Importance in Projection (VIP) scores.^53^ The top 100 VIP scoring genes, either positively or negatively correlated with the first component of the model, were used as input for gene set enrichment analyses. The enrichment was performed using the Gprofiler package, focusing on Gene Ontology categories for Biological Process, Molecular Function, and Cellular Component.^42^^,^^43^^,^^125^

Orthogonal validation was conducted for the PLSR models predicting cell count and motility. This involved analyzing publicly available datasets containing RNA-seq data paired with proliferation rates from 34 breast cancer cell lines^67^ and motility estimates from 28 breast cancer cell lines. We input the log2-normalized gene abundance data from these cell lines into our models to predict cell count or motility for each line. Pearson correlation was then calculated to compare the predicted phenotypes to the experimentally determined metrics.^54^

Additionally, we validated the PLSR model predicting cell count using the Project Achilles dataset for breast cancer cell lines from the Cancer Dependency Map Portal (DEPMAP).^63^^,^^64^ We compared VIP scores to Gene Effect scores calculated by DEPMAP from CRISPR screens and investigated the statistical significance of the relationship between VIP and Gene Effect scores using chi-squared analysis.

All single-cell RNAseq analysis of murine mammary epithelial cells was performed using Seurat^123^ on publicly available data.^68^ RNAseq generated from adult BL6, embryonic E18, and puberty ductal mammary epithelial cells were integrated, scaled, normalized, clustered, and then visualized with UMAP dimensional reduction. Expression of PLSR signatures were examined by calculating module scores using the AddModuleScore function. Differential expression analysis was performed using the FindMarkers function using default parameters.

#### Drug inhibition experiments

Cells were cultured in 24-well plates following previously established protocols. After cell attachment and subsequent culture in assay media, 3 nMol Trametinib (Selleckchem, #S2673), 1 μMol Alpelisib (Selleckchem, #S2814), or 5 nM AZD5069 (MedChemExpress, #19855) or DMSO was added along with the ligand treatments. Cell imaging and motility assessments were performed as previously described. To determine the statistical significance of changes in motility, we first fitted an ordinary least squares linear model to the data using the estimatr package in RStudio.^126^ Then, we estimated marginal means with the emmeans package and computed pairwise contrasts to compare motility across all ligand conditions for inhibitor-treated versus DMSO-treated cells.^127^

#### Reverse phase protein array

Cells were lysed and collected by manual scraping into 50-100 μL of lysis buffer (1% Triton X-100, 50 mM HEPES pH 7.4, 150 mM NaCl, 1.5 mM MgCl2, 1 mM EGTA, 100 mM Na pyrophosphate, 1 mM Na3VO4, 10% glycerol, 1x complete EDTA-free protease inhibitor cocktail (Roche #11873580001), 1x PhosSTOP phosphatase inhibitor cocktail (Roche #4906837001)). The lysates were incubated on ice for 20 minutes, followed by centrifugation at 14,000 rpm for 10 minutes at 4°C. The supernatant was collected, quantified using a BCA assay, and then mixed with 4X SDS sample buffer (40% glycerol, 8% SDS, 0.25 M Tris-HCl, 10% β-mercaptoethanol, pH 6.8). The mixture was boiled for 5 minutes and stored at -80°C. Three sets of biological replicates were submitted for RPPA testing. The samples underwent standard pre-processing using protocols established at the MD Anderson Cancer Center RPPA core.^128^ The RPPA data used in this study were part of a larger panel of conditions and perturbations assessed in MCF10A cells. The full dataset is available in Table S2.

#### siRNA experiment and QPCR

Cells were seeded in growth media at a density of 25,000 cells per well in a 6-well plate. Seven hours after seeding, the cells were transfected with either a commercially validated siRNA pool targeting CREB1 (Horizon Discovery #L-003619-00-0005) or a negative control siRNA (Horizon Discovery #L-003619-00-0005) using Lipofectamine RNAiMAX Transfection Reagent (Invitrogen #13778100) at a concentration of 25 nM siRNA. After 48 hours, the cells were treated with EGF, OSM, or a combination of EGF and OSM for an additional 24 hours. Five biological replicates for each treatment condition were collected.

To evaluate the changes in mRNA levels following siRNA-mediated CREB knockdown, we extracted total RNA from treated cells using the RNeasy Mini Kit (Qiagen # 74104) as per the manufacturer’s protocol. We synthesized cDNA using the iScript cDNA Synthesis Kit (Bio-Rad #1708891). The mRNA expression levels were quantified through real-time qPCR with SYBR green chemistry on the Bio-Rad CFX Opus 384 Real-Time PCR System (Bio-Rad #12011452). The results were normalized to Glyceraldehyde 3-phosphate dehydrogenase (GAPDH) levels using the 2-ΔΔCt method.^129^ The raw QPCR dataset is available in Table S4.

The primer sequences for the target mRNAs are provided in Table S3.

#### ELISA

Conditioned media were collected in triplicate from MCF10A cells treated with EGF or EGF+OSM for 24 hours following siRNA-mediated knockdown of CREB or non-targeting control. Media were cleared by centrifugation and stored at –80°C until analysis. CXCL3 and CXCL5 protein levels were quantified using commercially available ELISA kits (AFG Scientific, #EK712056 #EK712055) according to the manufacturer’s instructions. Absorbance was measured at 450 nm using a GloMax Explorer (Promega, #GM3500) microplate reader and concentrations were calculated based on standard curves generated in duplicate. Raw absorbance measurements are available in Table S5.

### Quantification and statistical analysis

All statistical analyses were performed in RStudio.^119^ For all statistical tests, a *p*-value < .05 was considered significant.

#### Phenotypic comparisons

Ligand perturbation experiments were run in triplicate. To assess the statistical significance of the deviation in combination ligand phenotypes from single ligand conditions, ANOVA was employed followed by Tukey’s Honest Significant Differences test for post-hoc comparisons. For phenotype comparisons with EGF and PBS, ANOVA followed by Dunnett’s test for post-hoc comparisons was performed using the DescTools package.^130^

Motility experiments were run in triplicate. To determine the statistical significance of changes in motility, we first fitted an ordinary least squares linear model to the motility quantifications using the estimatr package in RStudio.^119^ Then, we estimated marginal means with the emmeans package and computed pairwise contrasts to compare motility across all ligand conditions for inhibitor-treated versus DMSO-treated cells.^127^ *p*-values were adjusted using Tukey’s method for multiple comparisons.

#### Molecular comparisons

Significantly induced genes were defined as genes with a LFC > 1.5 or LFC < -1.5 and a *p*-value < .05, adjusted using the Benjamini and Hochberg method.^131^ Statistical comparisons of differentially expressed genes within each module were performed using chi-squared analysis, followed by examination of standardized residuals. We compared VIP scores to Gene Effect scores calculated by DEPMAP from CRISPR screens and investigated the statistical significance of the relationship between VIP and Gene Effect scores using chi-squared analysis. Statistical significance in RPPA antibody intensity between the EGF+OSM condition and both single ligand conditions was determined using ANOVA followed by Dunnett’s test using the DescTools package.^130^ Statistical comparison in expression levels between the EGF+OSM siCREB1 condition and the EGF+OSM siSCR condition was determined by student’s T-test performed on ΔCt values or ELISA protein concentrations estimated from a fitted standard curve.^132^

## Supplemental information

Document S1. Figures S1–S5 and Table S3

Table S1. LC-MS/MS mass charge ratios related to Figure 1

Table S2. RPPA intensity values related to Figures 6 and S5

Table S4. QPCR Quantification Cycle values related to Figures 6 and S5

Table S5. ELISA absorbance values related to Figure 6
