# Supplementary material for: Microenvironmental signals combine to induce non-additive molecular and phenotypic responses in mammary epithelial cells
Source: iScience. 2025 Aug 20;28(9):113407. doi: 10.1016/j.isci.2025.113407 (PMC12496203; doi:10.1016/j.isci.2025.113407)
Supplement: Document S1. Figures S1–S5 and Table S3 [file mmc1.pdf]

iScience, Volume *28*

Supplemental information

Microenvironmental signals combine to induce non-additive molecular and phenotypic responses in mammary epithelial cells

Ian C. McLean, Sean M. Gross, Jeremy Copperman, Daniel S. Derrick, Indranil Paul, Andrew Emili, and Laura M. Heiser

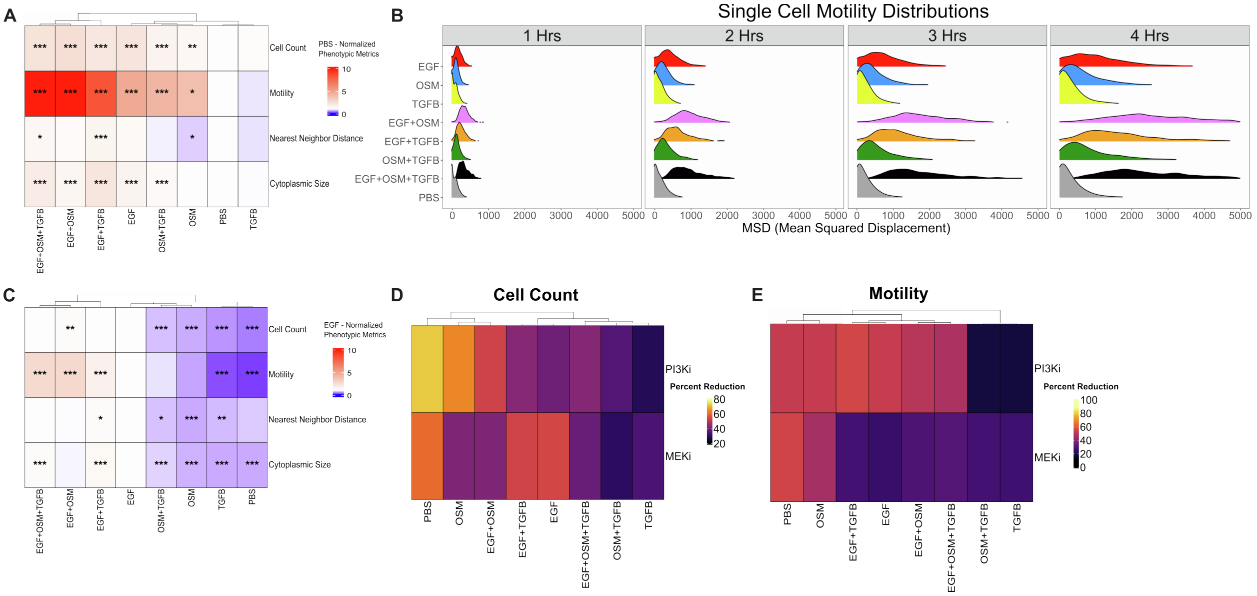

Figure S1: Single ligand proteomic comparison and statistical comparison of phenotypic responses. Related to Figure 1.

(A) Quantified phenotypic metrics for all ligand treatments normalized by and compared to PBS. Statistical significance was determined using Dunnett's test with a p-value <.05 considered significant. $*p < 0.05$, $**p < 0.01$, $***p < 0.001$. (B) Single cell distributions of motility assessed through MSD quantification. EGF+OSM and EGF+OSM+TGFB exhibit a bimodal distribution of slower and faster moving cells. (C) Quantified phenotypic metrics for all ligand treatments normalized by and compared to EGF. Statistical significance was determined using Dunnett's test with a p-value <.05 considered significant. $*p < 0.05$, $**p < 0.01$, $***p < 0.001$. (D-E) Quantification of ligand induced phenotype after treatment with Trametinib (MEKi) or Alpelisib. Values shown represent the precent reduction in the phenotypic metric as compared to vehicle control for each ligand condition.

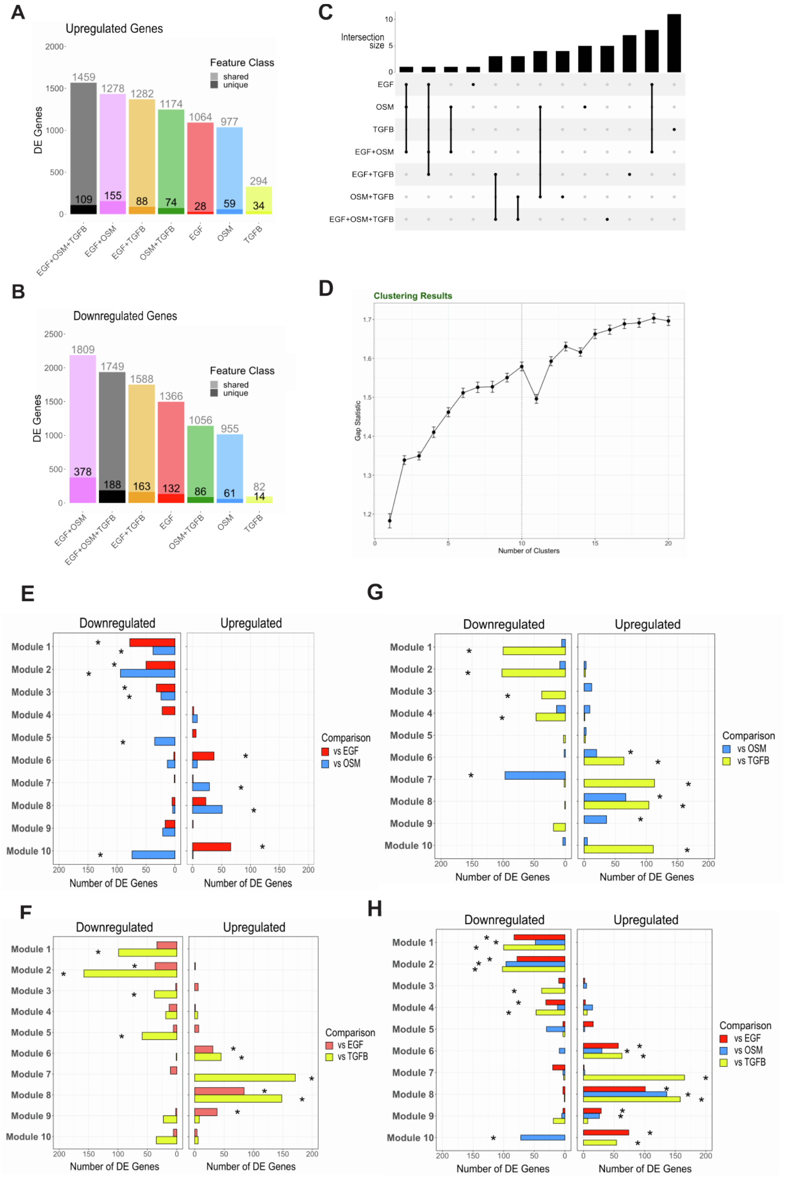

Figure S2: Detailed transcriptional analysis of single and combination ligand treatments. Related to Figure 2.

(A) Number of upregulated differentially expressed genes (LFC > 1.5, q-value < 0.05) for each treatment relative to PBS control. Genes unique to each treatment are shown with solid transparency, while those shared with at least one other condition are shown with lighter transparency. (B) Number of downregulated differentially expressed genes (LFC < -1.5, q-value < 0.05) for each treatment relative to PBS control, with transparency representing unique versus shared genes, as in panel A. (C) Upset plot showing the overlap of transcriptional regulators activated by single and combination treatments, highlighting the shared and unique regulatory programs across conditions. (D) Gap analysis identifies 10 optimal gene modules across treatments, based on clustering of transcriptional data. (E-H) Comparisons of gene module scores for each combination treatment versus the respective single ligand conditions. Statistical significance was determined by Chi-squared analysis, comparing the number of differentially expressed genes in each treatment relative to T0 for each module. P-value < .05 was considered significant. $*p < 0.05$.

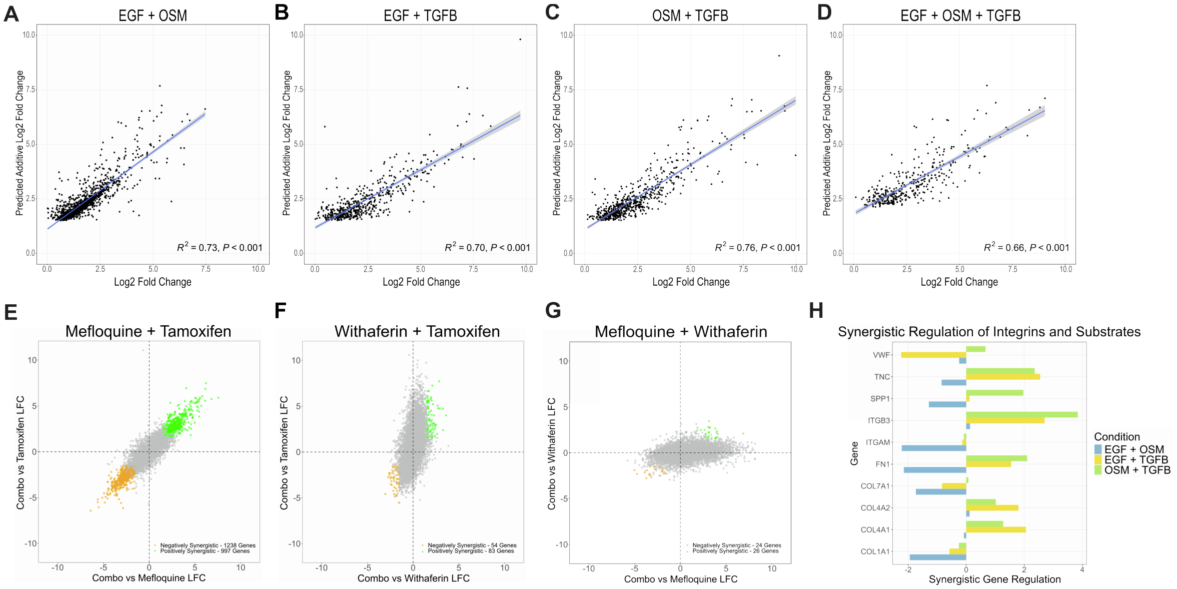

Figure S3: Additive modeling of ligand combinations and transcriptional synergy analysis of drug treatments. Related to Figure 3.

(A-D) Scatterplots depicting the correlation between the observed log2 fold change (LFC) for upregulated genes (LFC > 0.5) in each ligand combination condition (EGF+OSM, EGF+TGFB, OSM+TGFB, and EGF+OSM+TGFB) and the predicted LFC based on a purely additive model summing the LFC of the individual ligands. Pearson correlation coefficients (R^2) are reported for each comparison, demonstrating strong correlations (R^2 values ranging from 0.66 to 0.76, p-value < .001), indicating that ligand combination responses are predominantly additive. Shaded region represents 95% confidence interval. (E-G) Transcriptional synergy analysis applied to a published dataset of MCF7 cells treated with Tamoxifen, Mefloquine, and Withaferin individually and in combination. Scatterplots highlight the number of synergistic genes (positive and negative) induced by each drug combination, providing a comparison to ligand-induced transcriptional synergy. (H) Integrin receptors and substrates synergistically regulated by combination treatments.

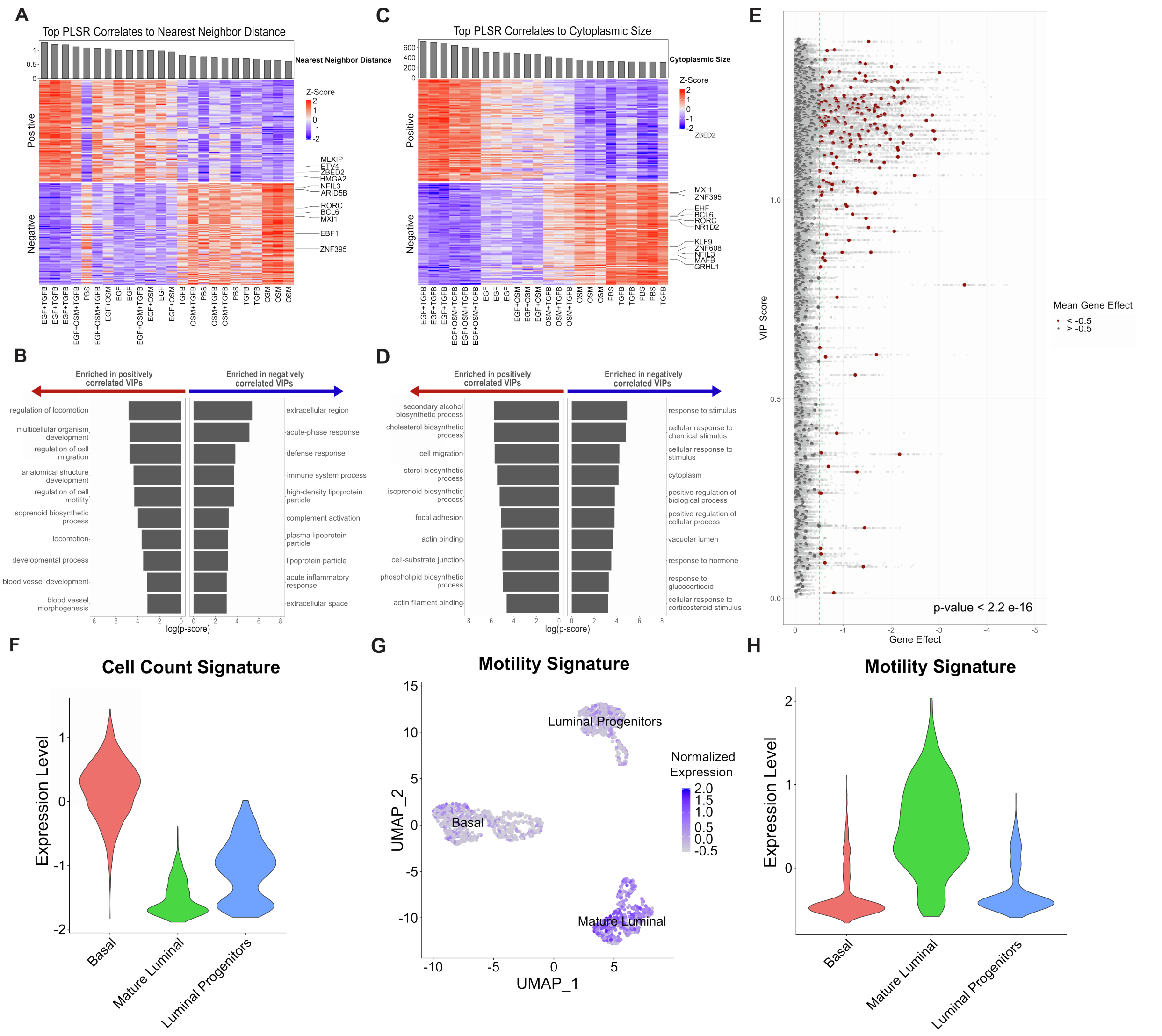

Figure S4: PLSR signature GSEA and expression in developing murine mammary ductal epithelial cells. Related to Figure 4.

(A) Z-scored expression of the top VIP genes associated with the Nearest Neighbor Distance phenotype in the PLSR model. (B) Gene set enrichment analysis of the top VIP genes associated with Nearest Neighbor Distance. (C) Z-scored expression of the top VIP genes associated with Cytoplasmic Size from the PLSR model. (D) GSEA of the top VIP genes associated with Cytoplasmic Size. (E) Evaluation of the Cell Count PLSR model using the DEPMAP dataset, where all genes in the model were analyzed for their Gene Effect scores across breast cancer cell lines. VIP genes that were positively correlated with cell count exhibited significantly lower Gene Effect scores (χ^2 = 902.41, p-value < 2.2e-16), supporting their role in regulating cell viability and proliferation. (F) Expression of the Cell Count PLSR gene signature is enriched in basal cells. (G) UMAP representations of murine mammary ductal epithelial cells during puberty. Expression of the Motility PLSR gene signature is enriched in mature luminal cells. (H) Expression of the Motility PLSR gene signature across mammary epithelial subtypes.

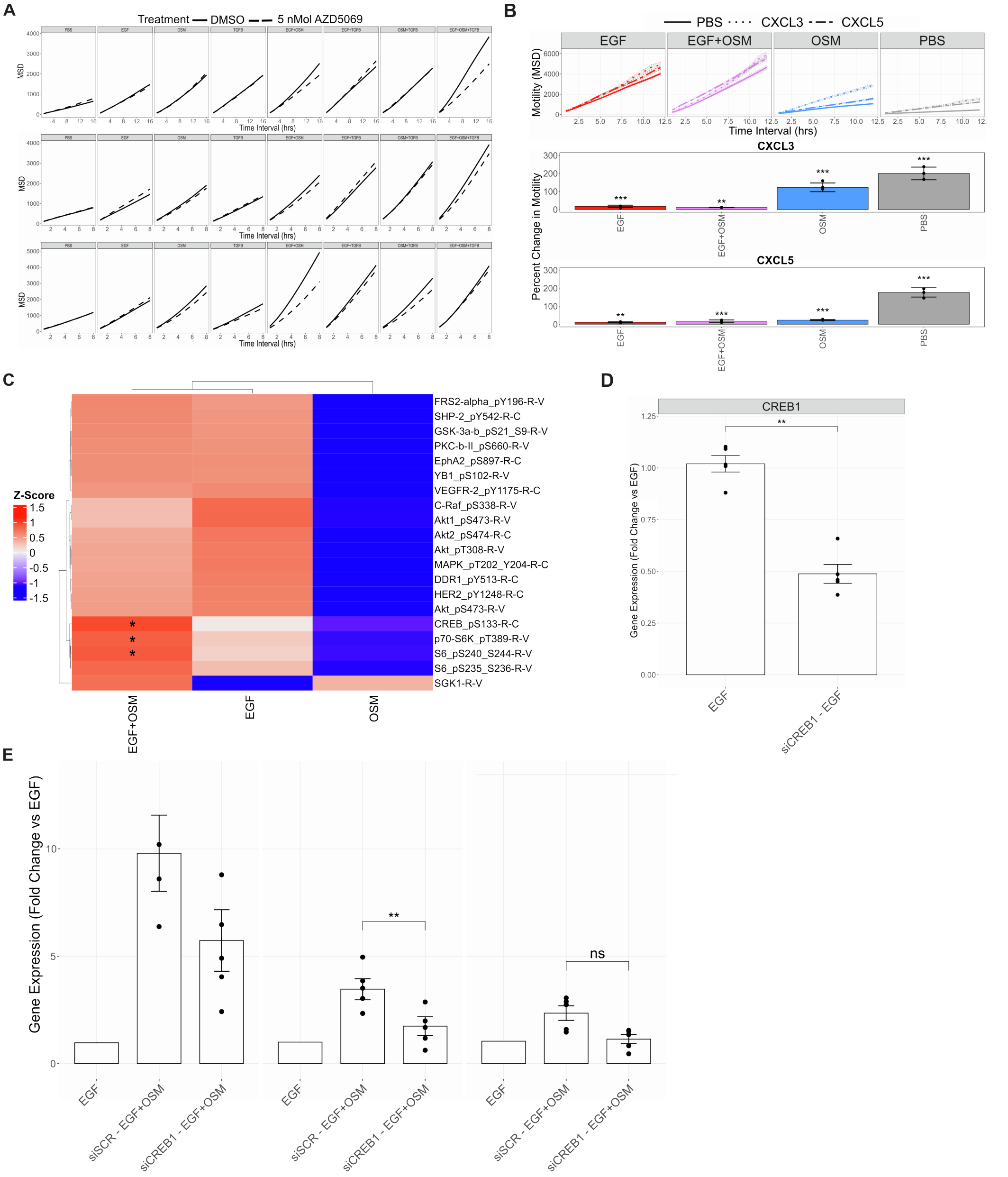

Figure S5: Cell Motility Assays, CREB Knockdown Efficiency, and RPPA Data. Related to Figure 6.

(A) Cell motility assay data showing the effects of CXCR2 inhibition (AZD5069) on MCF10A cell motility in the EGF+OSM, EGF, and OSM conditions, across all three biological replicates. The data depict the mean squared displacement, with CXCR2 inhibition significantly reducing motility in the EGF+OSM condition. (B) Cell motility assays following treatment with select ligand panel in the presence of exogenous 5ng/mL CXCL3, CXCL5, or PBS. Exogenous CXCL treatment increased motility in all ligand conditions. Data shown as median change in motility and error bars representing the standard deviation from three biological replicates. ANOVA followed by post-hoc Tukey's honest significant difference test was used to assess significance, with p-value < 0.05 considered significant (n=3). $*p < 0.05$, $**p < 0.01$, $***p < 0.001$. (C) Reverse Phase Protein Array (RPPA) data from cells treated with EGF, OSM, and EGF+OSM. Dunnett's test was used to compare the single ligand conditions to the EGF+OSM combination, revealing statistically significant changes (p-value < .05) in CREB activation in the EGF+OSM condition (n=3). $*p < 0.05$. (D) qPCR confirmation of CREB knockdown in MCF10A cells. Barplot shows mean CREB expression in EGF treated cells with siCREB1 or siSCR. Error bars indicate 95% confidence interval (n=5). Student's t-test was used to assess significance. $*p < 0.05$, $**p < 0.01$, $***p < 0.001$. (E) qPCR analysis of chemokine expression in CREB knockdown cells following EGF+OSM treatment. Expression of chemokines under the EGF control condition is included for reference. Barplots depict mean fold change in expression compared to

the EGF control, with error bars representing the 95% confidence interval. Student's t-test was used to assess significance (n=5). $*p < 0.05$, $**p < 0.01$, $***p < 0.001$.

Table S3: QPCR Primer Information

Name	Sequence	Scale	Purification	Primer_ID
CREB1_FWD	CCACTGTAACGGTGCCAACT	25nm	STD	215490111c2
CREB1_REV	GCTGCATTGGTCATGGTTAATGT	25nm	STD	
CXCL3_FWD	CGCCCAAACCGAAGTCATAG	25nm	STD	4504157a1
CXCL3_REV	GCTCCCCTTGTTCAGTATCTTTT	25nm	STD	
CXCL5_FWD	AGCTGCGTTGCGTTTGTTTAC	25nm	STD	296080750c1
CXCL5_REV	TGGCGAACACTTGCAGATTAC	25nm	STD	
PPBP_FWD	GTAACAGTGCGAGACCACTTC	25nm	STD	41872613c1
PPBP_REV	CTTTGCCTTTCGCCAAGTTTC	25nm	STD	
GAPDH_FWD	GGAGCGAGATCCCTCCAAAAT	25nm	STD	378404907c1
GAPDH_REV	GGCTGTTGTCATACTTCTCATGG	25nm	STD	
